# BRENTUXIMAB VEDOTIN AND BENDAMUSTINE (BvB) IN PATIENTS WITH RELAPSED OR REFRACTORY HODGKIN LYMPHOMA: AN INTERNATIONAL, MULTICENTER, SINGLE-ARM, PHASE 1–2 TRIAL

**DOI:** 10.1016/S1470-2045(17)30912-9

**Published:** 2017-12-21

**Authors:** Owen A. O’Connor, Jennifer K. Lue, Ahmed Sawas, Jennifer E. Amengual, Changchun Deng, Matko Kalac, Lorenzo Falchi, Enrica Marchi, Turenne Ithamar, Renee Lichtenstein, Celeste Rojas, Mark Francescone, Lawrence Schwartz, Bin Cheng, Kerry J. Savage, Diego Villa, Michael Crump, Anca Prica, Vishal Kukreti, Serge Cremers, Joseph M. Connors, John Kurvuilla

**Affiliations:** 1 Center for Lymphoid Malignancies, Department of Medicine, Columbia University Medical Center – College of Physicians and Surgeons, New York, N.Y.,; 2 Department of Radiology Columbia University Medical Center – College of Physicians and Surgeons, New York, N.Y.,; 3 Department of Biostatistics, Mailman School of Public Health, Columbia University Medical Center – College of Physicians and Surgeons, New York, N.Y.,; 4 Clinical Translational Research Center, Laboratory of Analytical Pharmacology, Columbia University Medical Center – College of Physicians and Surgeons, New York, N.Y.,; 5 BC Cancer Agency, Centre for Lymphoid Cancer Vancouver, BC, Canada,; 6 Princess Margaret Cancer Centre, Toronto, ON, Canada

## Abstract

**Background:**

The major objective of this study was to explore the safety and clinical activity of Brentuximab vedotin (Bv) and bendamustine in combination in patients with relapsed or refractory Hodgkin Lymphoma. Bv produces high response rates and durable progression-free survival (PFS) in CD30-expressing lymphomas and is approved for the treatment of relapsed Hodgkin lymphoma (HL) and relapsed ALCL. Bendamustine (B) is active agent across the lymphoproliferative malignancies, though the PFS among patients with HL and PTCL is modest.

**Methods:**

This was an international, multicenter, single-arm, Phase 1–2 study of BvB in patients with relapsed or refractory HL and ALCL. Eligible patients were required to have relapsed/refractory CD30+ biopsy proven HL or ALCL and an ECOG Performance Status ≤2. In the Phase 1, HL patients were deemed eligible if they developed progressive disease following or after declining ASCT, or had at least 2 prior multi-agent chemotherapy regimens. In the Phase 2, patients with HL were eligible if they had relapsed or refractory disease after one line of therapy. Eligible ALCL patients were required to have relapsed after at least one prior multi-agent chemotherapy regimen and if they were not eligible for or have declined ASCT. The primary objective of the Phase I portion of this study was to identify the maximum tolerated dose (MTD) and dose limiting toxicity (DLT). The primary endpoint of the Phase 2 portion was to determine the overall response rate (ORR; complete response [CR] plus partial response [PR])) based on an intention to treat analysis (ITT). Secondary objectives of Phase 1–2 included assessing for duration of response, progression free survival and overall survival. Response was evaluated using International Harmonization Project Group 2007 Revised Response Criteria. Bv was escalated from 1.2mg/kg Day 1, and B from 70mg/m2 Days 1 and 2 every 21 days until the MTD or recommended phase 2 dose (RP2D) was reached. The study is ongoing but no longer recruiting patients. This trial is registered with ClinicalTrials.gov number NCT01657331.

**Findings:**

65 patients (only 1 ALCL) were treated, 28 on the Phase 1 and 37 on the phase 2. While the MTD of the combination was not reached, the single agent MTD of Bv (1.8mg/kg Day 1) and RP2D of B (90mg/m2 Days 1 and 2) were identified as the RP2D of the combination. Patients were heavily treated, 65% (42 of 65) had an autologous or allogeneic stem cell transplant or both. The Phase 1 revealed modest toxicity. The major Grade 3/4 toxicities included Grade 3 lung infection in 5 (14%) patients in the Phase 2, and Grade 3/4 neutropenia in 13 (24%) patients across the Phase 1 and 2. The Phase 1 and 2 overall response rates (ORR) were 61% and 78% respectively, with 43% (16 of 27) patients treated in the Phase 2 attaining a complete remission (CR). In the Phase 2, the median PFS has not been reached and duration of response (DOR) was 3.4 months. There was a total of 23 deaths with 21 due to progression of disease, 2 occurring after being transplanted, and none of which were treatment related.

**Interpretation:**

This demonstrates that BvB might be an effective salvage regimen for patients with HL, with a favorable safety profile.

**Funding:**

Seattle Genetics, The Lymphoma Research Fund of Columbia University and National Center for Advancing Translational Sciences, National Institutes of Health, through Grant Number **UL1TR001873** provided support for this investigator initiated sponsored trial. The content is solely the responsibility of the authors and does not necessarily represent the official views of the NIH.

## INTRODUCTION

Hodgkin lymphoma (HL) and anaplastic large T-cell lymphoma (ALCL) are the two subtypes of lymphoid malignancies characterized by robust expression of CD30. CD30 is one of the defining cell surface markers on the Reed-Sternberg cell in patients with HL, and ALCL. Bv is an antibody drug conjugate (ADC), where the antibody is a chimeric IgG1 directed against CD30 which is covalently attached via a linker to a microtubule disrupting agent, monomethyl auristatin E (MMAE).

Bv was approved by the U.S. Food and Drug Administration (FDA) for the treatment of patients with Classical HL after failure of autologous stem cell transplant (ASCT) or failure of at least two prior multi-agent chemotherapy regimens or in patients who are not ASCT candidates. Results from 102 patients with HL treated on the pivotal Phase II study demonstrated an ORR of 73%, with 32% attaining CR^1^. The median progression free survival (mPFS) for the population was 5.6 months, though for patients in CR, the mPFS was 21.7 months. Patients achieving a PR or less did not experience a PFS advantage relative to the study population. Bv has also been approved for patients with ALCL after failure of at least one prior multi-agent chemotherapy regimen, with an ORR of 86%, and a CR rate of 57%, with a mPFS for the population and patients in CR being 13.3 months and 14.6 months respectively^2^.

Bendamustine (B) is a fusion hybrid molecule containing the purine analog fludarabine and the alkylating nitrogen mustard mechlorethamine. In patients with HL (n = 34), B produced an ORR of 53% with 33% of patients achieving CR, and a mPFS of 5.2 months^3^. While the BENTLY study evaluated B in patients with relapsed or refractory PTCL, there is no dedicated experience with B in patients with ALCL^4^.

Collectively, these data suggest that combination therapy employing Bv and B could achieve several important goals. First, it could improve the CR rate seen with either single agent, which could deepen remissions leading to improved PFS and DOR. As CR prior to ASCT is the single most important determinant of prognosis for these patients, then improving this particular metric could translate into more effective consolidation strategies. Second, a two-drug combination could reduce the chemotherapy related toxicity burden compared to standard platinum or gemcitabine based salvage regimens including ICE (ifosfamide, carboplatin, etoposide)^5^, DHAP (cisplatin, cytarabine and dexamethasone)^6^, or gemcitabine based regimens like GND (gemcitabine, vinorelbine and doxil)^7^, GemOx (gemcitabine and oxaliplatin)^8^, GDP (gemcitabine, dexamethasone, and cisplatin)^9^, IGEV (ifosfamide, gemcitabine, etoposide and vinblastine)^10^ and BeGEV (bendamustine, gemcitabine, etoposide and vinblastine)^11^. Finally, this combination could shift typically inpatient based therapy to the outpatient arena, reducing costs of hospitalization. We also conducted an extensive biomarker discovery initiative to identify possible predictive biomarkers. We initiated an international, multicenter, single-arm, Phase 1 – 2 study of BvB in patients with relapsed/refractory HL and ALCL to explore the merits of the combination.

## METHODS

### Study design and participants

The Institutional Review Boards (IRB) of all participating center approved the study, which was conducted according to the provisions of the Declaration of Helsinki and the International Conference on Harmonization Guidelines for Good Practice. This was an open-label, phase 1–2, international multicenter study registered with ClinicalTrials.gov (identifier NCT01657331). The complete version of the protocol is available in the supplementary webappendix (pages 11–63). All patients enrolled provided written informed consent. All authors had access to primary data.

Eligible patients were required to have relapsed/refractory CD30+ biopsy proven HL or ALCL. In the Phase 1, HL patients were deemed eligible if they developed progressive disease following or after declining ASCT, or had at least two prior multi-agent chemotherapy regimens (if they are not ASCT candidates). In the Phase 2, patients with HL were eligible if they had relapsed or refractory disease after one line of therapy. Eligible ALCL patients were required to have relapsed after at least one prior multi-agent chemotherapy regimen and if they were not eligible for or have declined ASCT. Patients were required to have measurable or evaluable disease (Phase 1 only), ECOG performance status ≤2, were ≥ 18 years, and documented adequate organ and marrow function defined as follows: ANC > 1000; platelet count > 50,000; liver function tests (AST/ALT) < 2.0 x institutional upper limit of normal; total bilirubin < 1.5 x institutional limit; creatinine clearance > 50mL/min. Patients who had relapsed after ASCT and/or allogeneic SCT (AlloSCT) were also eligible. Patients had an estimated life expectancy of 6 months or more.

Patients were excluded if they were previously treated with Bv and B in combination, but were eligible if they had received either as a single agent. Systemic steroids were required to be stabilized to ≤10 mg/day 7 days prior to the initiation of trial. Patients with known cerebral or meningeal disease, active concurrent malignancy, pre-existing neuropathy grade 3 or greater, HIV, hepatitis B, or C, were pregnant or nursing or had uncontrolled intercurrent illness were also ineligible.

Response was assessed in accordance with the International Harmonization Project Group 2007 Revised Response Criteria^12^.

### Procedures

**Figure 1.**
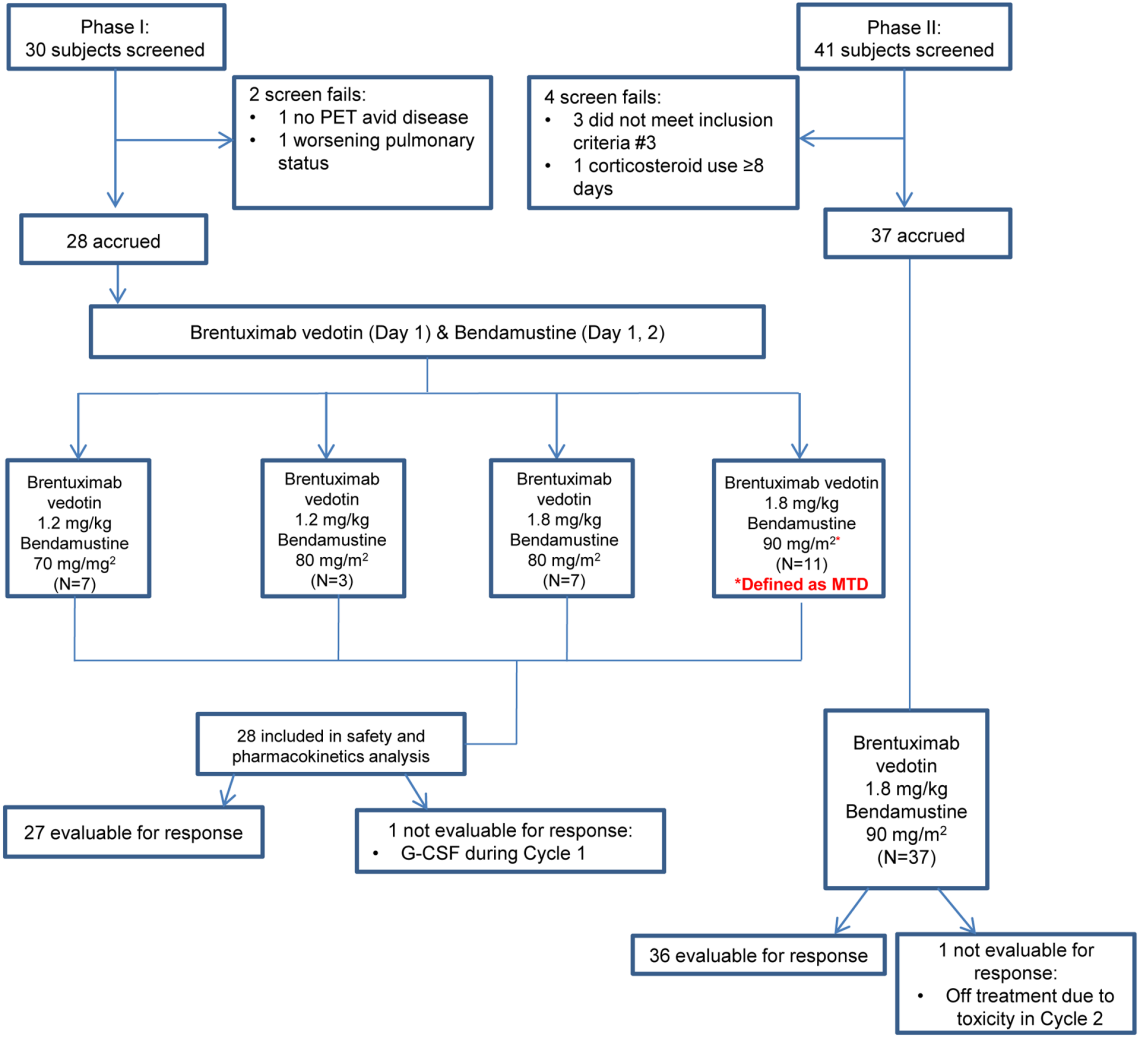
Schematic of Patient Disposition in the Phase 1 and 2 Schema depicts the complete accrual of all patients to the Phase 1 and 2 portions of the study, describing dose cohorts, screen failures, and number of patients evaluable for safety and response.

The study was conducted at three institutions: Columbia University Medical Center, N.Y; and two sites in Canada including the British Columbia Cancer Agency (BCCA) in Vancouver, and Princess Margaret Cancer Centre in Toronto. The trial design and patient disposition is depicted in Figure 1. Dose escalation in the phase 1 proceeded according to a standard 3 + 3 dose escalation design with no intra-cohort dose escalation. Bv was dosed on Day 1, while B was dosed on Days 1 and 2, on a 21 days cycle. Four dose cohorts were enrolled as shown in Figure 1, starting at a dose of 1.2mg/kg for Bv escalating to a dose of 1.8mg/kg. The starting dose of B was 70mg/m^2^ and was escalated to 90 mg/m2. Patients were eligible to receive prophylactic pegfilgrastim on day 3 of any subsequent cycle after cycle 1 or filgrastim for 5 to 10 days per investigator’s discretion, which was not captured in the case report form (CRF). Cohorts were expanded by 3 patients until the MTD was met. DLT were limited to Cycle 1 events only. A maximum of 6 cycles was allowed, and no dose reductions were permitted. After cycle 1, dose delays up to 3 weeks were allowed, while delays of more than 3 weeks led to study discontinuation. All adverse events were identified using the Common Toxicity Criteria version 4.0, and were monitored at all routine visits and with each treatment. Laboratory evaluation was performed on day 1 of each cycle, and routinely included CBC with differential, comprehensive profile, lactate dehydrogenase (LDH) and erythrocyte sedimentation rate (ESR). Blood for biomarkers was obtained pretreatment, and then on day 1 and 8 of Cycle 1, then Day 1 of Cycle 2, 3 and at end of treatment (within 14 days of last dose). Standard supportive treatments were allowed as clinically indicated including anti-emetics, antipyretics, antihistamines, analgesics, antibiotics, antivirals, and blood products.

### Definitions of dose-limiting toxicities, by grade

- Any missed dose within cycle 1 and/or toxicity that is possibly related to drug, occurring up to 7 days after completion of cycle 1 that results in a delay of initiating cycle 2
- Grade 4 neutropenia that does not resolve to ≤ Grade 2 within ≤ 7 days
- Grade 4 thrombocytopenia lasting more than 7 days
- Grade 3 febrile neutropenia (ANC<1000/mm^3^ with a single temperature of > 38.3°C or sustained temperature of ≥ 38°C for over one hour)
- Any Grade ≥ 3 non-hematologic toxicity, with the specific exception of:
  - Nausea, vomiting, diarrhea, or dehydration lasting > 48 hours in the setting of inadequate compliance with supportive care measures
  - Grade 3 hypercholesterolemia, hypertriglyceridemia, constipation and fatigue

Responses were investigator assessed, and not centrally reviewed. They were determined using clinical parameters and computed tomography or positron emission tomography/computed tomography (CT/PET). Efficacy was evaluated after cycle 2 and 6; thereafter, tumor assessment was performed clinically every 3 to 6 months until progression of disease (POD) and/or subsequent therapy. Patients were removed from study if they had disease progression, untoward toxicity, or voluntarily withdrew consent. Efficacy analyses was based on an intention to treat (ITT) analysis, and all patients were followed for survival until disease progression or new therapy is initiated.

### Outcomes

The primary endpoint of the Phase 1 was to determine the DLT (as defined above) and MTD (defined as the highest dose level at which <33% of the dose cohort (0 of 3 or 1 of 6) experience a DLT in the first cycle of therapy) of BvB, while the secondary endpoints were to determine the ORR (CR plus PR), PFS (defined as the time from randomization until disease progression or death due to any cause, whichever occurred first) and DOR (defined as the time from documentation of a response to treatment to the first documentation of tumor progression or death due to any cause whichever comes first). In the Phase 2, the primary endpoint was investigator assessed overall response, defined as the percentage of patients who achieved a confirmed complete or partial response and the secondary endpoints were DOR, PFS and Overall Survival (OS was described as the time from randomization to the date of death due to any cause or last date of contact).

Detailed exploratory correlative studies were also assessed. While TARC measurement was part of a preplanned analysis, sCD30, CD163 and galectin were not planned in the original protocol. These markers were studied given the abundance of literature suggesting their potential as predictive biomarkers in varying situations. sCD30 was quantified in serum samples using an enzyme–linked immunosorbent assay (ELISA) kit from eBioscience (San Diego, CA, USA) with an intra-assay coefficient of variation (CV) of 4% and inter-assay CV of 5.62%. CD163 was assessed in serum samples with a quantikine ELISA kit from R&D systems (Minneapolis, MN, USA). The intra-assay precision was 3.57% and inter-assay CV was 5.07%. Serum samples were also assessed for galectin-1 by ELISA (R&D systems, Minneapolis, MN, USA; intra-assay CV% 7.2 and inter-assay CV 8.6%) and CCL17/Thymus and Activation regulated chemokine (TARC) using an ELISA kit from R&D systems (Minneapolis, MN, USA; intra assay CV 4.03% and inter-assay CV 8.23%) according to manufacturer’s instructions.

### Statistical analysis

All patients who completed at least one cycle of therapy and had at least one restaging visit were evaluable for response and all time-to-event analyses. All patients for whom we had appropriate quantities of blood, irrespective of outcome, were included in the biomarker studies. For the Phase 2, a two-stage Simon Minimax design was used with a first stage that included 18 patients, with a total of 37 patients. The two stage design tests the hypothesis that the ORR is less than or equal to 0.50 versus the alternative that the ORR is greater than or equal to 0.75.

OS and PFS were estimated using Kaplan-Meier method and group comparison were assessed using two-sided log-rank test and Cox regression for estimating the hazard ratios (95% CI). Due to sample size considerations, group comparison of biomarkers was based on the two-sample t-test. Continuous variables, which includes all biomarker variables, were summarized as mean +/− SD while categorical variables as count (%). All the analyses were performed in SAS (v. 9.4, Cary, NC, USA), using a type I error of 0.05. No multiple testing adjustments were performed as this part of the analysis is exploratory. This study is registered with ClinicalTrials.gov number NCT01657331.

### Role of the funding source

This was an investigator sponsored study supported in part by Seattle Genetics. Teva provided bendamustine. The sponsors played no role in designing the study, collecting or analyzing data, or in preparing the manuscript. The study was designed by the authors and data was collected by study site staff and monitored by Columbia University staff. Data was analyzed by the authors, and the manuscript written by and approved by all authors. All authors had full access to all data in the study, and had final responsibility for the decision to submit for publication. The corresponding author had full access to all of the data and the final responsibility to submit for publication.

## RESULTS

Figure 1 shares the disposition for all patients screened and enrolled (between July 26, 2012 to May 31, 2017). In the Phase 1, 30 patients were screened, with 2 screen fails and 28 patients accrued. Dose escalation proceeded as shown, with the doses of 1.8mg/kg of Bv and 90mg/m2 of B being declared the recommended Phase 2 dose (RP2D). No MTD was actually attained, so escalation was stopped at the single agent MTD of Bv, and the RP2D used for B, confirming no requirement for attenuation of dose for these agents in this combination. A Dose Level 5 of Bv at 1.8mg/kg and B at 120mg/m2 was planned originally. This cohort was not accrued because we were seeing manageable toxicity with good activity at the Phase 2 doses routinely used in the clinic for the single agents. Hence, the Study Investigators collectively decided not to escalate given concerns about excess toxicity. All 28 patients were eligible for the safety analysis, and 27 for response. In the Phase 2, 41 patients were screened with 37 accrued, with 4 screen fails as described in Figure 1. Of the 37 patients, 36 were evaluable for response, as one patient came off study in Cycle 2. Table 1 shares the demographic features of the study population. Of note, only one patient had ALCL and was treated on the Phase 1. The median age of the Phase 1 and 2 populations were 38 and 34 respectively, while 18 of 28 (64%) and 23 of 37 (62%) of the patients were male respectively.

The population was very heavily treated, with a median number of prior therapies being 5 (range 2–12) and 3 (1–8) in the Phase 1 and 2 respectively. The individual treatment history of patients enrolled on the Phase 1 and 2 as a function of dose cohort, with the observed DLT, a co-primary endpoint for the Phase I, and their clinical outcome is presented in Supplemental Tables 1 and 2 (webappendix page4–5). The majority of patients had undergone some form of stem cell transplant. Twenty-one of 28 (75%) and 21 of 37 (27%) had undergone an ASCT in the Phase 1 and 2 respectively, while 2 of 28 (7%) and 1 of 37 (3%) had undergone an AlloSCT in the Phase 1 and 2 respectively. Despite being so heavily treated, the combination was very well tolerated. Table 2 depicts the MedDRA toxicity occurring in ≥ 10% of patients in both the Phase 1 and 2. While no Grade 4 toxicities were noted in the Phase 1, the most common Grade 3 toxicities included anemia (18%); thrombocytopenia (14%); neutropenia (11%) and infusion related reactions (7%). Interestingly, the spectrum of toxicity was different in the Phase 2, with the major toxicity being neutropenia (Grade 3 and 4 in 27% and 8% respectively), and lung infection (14%), possibly a reflection of lesser prior therapy in the Phase 2 cohort. The most common Grade 1 or 2 toxicities seen in the phase 2 included nausea (68%), fatigue (68%), fever (38%), diarrhea and vomiting (38% each). Specific patient accounting as a function of dose cohort expanded is as follows: (1) in Cohort 1, 7 patients were accrued, 1 of whom had to be replaced due to voluntary withdrawal of consent, 1 experienced a DLT of neutropenia leading to an additional 3 patients enrolled; (2) in Cohort 2, 3 patients were enrolled without DLT; (3) in Cohort 3, 7 patients were enrolled, 1 had to be removed because they inadvertently received GCSF (a protocol violation), 1 experienced a DLT of neutropenia, leading to the addition of 3 patients with no further DLT; and (4) in Cohort 4, 11 patients were enrolled, 1 experienced a DLT of neutropenia leading to the addition of 3 patients, and 5 more at the RP2D per study (Figure 1). The MTD was not reached, based on the fact there was no maximum administrable dose identified. The RP2D was Bv at 1.8mg/kg and B at 90mg/kg. Only 1 of 11 patients qualified as a DLT (Grade 4 neutropenia) at the RP2D (i.e. Cohort 4). In Phase 2, the median number of cycles administered was 5 (range 2–6). There were 4 patients who had to discontinue drug due to toxicity, including 2 in the phase 1 (both at dose cohort 4, including one for an infusion related reaction, and one for an anaphylactic reaction), and 2 in the Phase 2 (one for kidney infection, one for dehydration).There were no dose reductions or treatment related deaths. To date, 23 of 65 patients have died, 21 of whom succumbed to disease progression, 2 of whom died from complications related to stem cell transplant (one autologous and one allogeneic).

**Figure 2.**
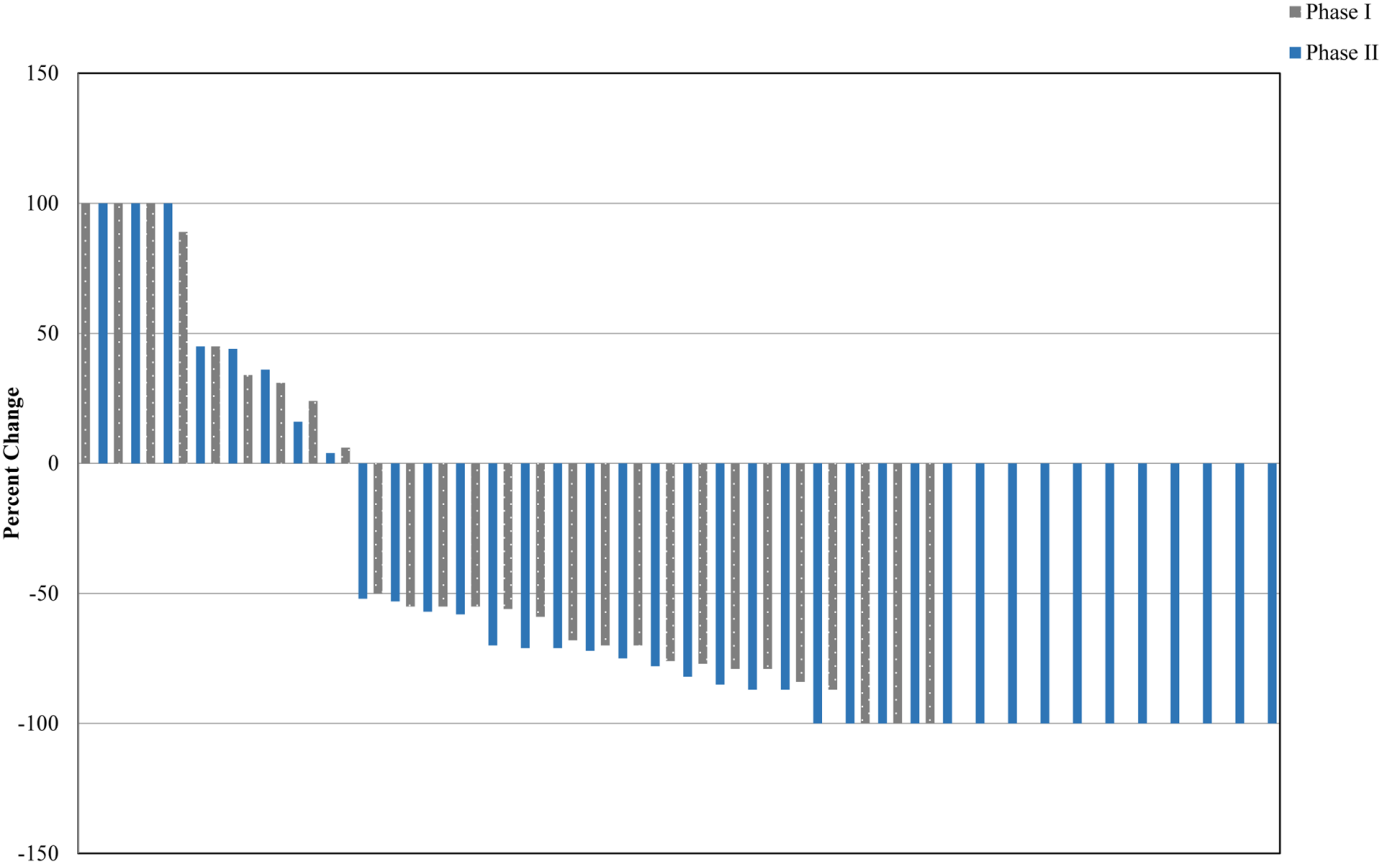
Waterfall Plot of Phase I and Phase II Patients Waterfall plot depicting responses for patients in the Phase 1 and Phase 2. Changes in tumor baseline are shown, with all complete remissions being defined by functional imaging as PET negative.

**Figure 3.**
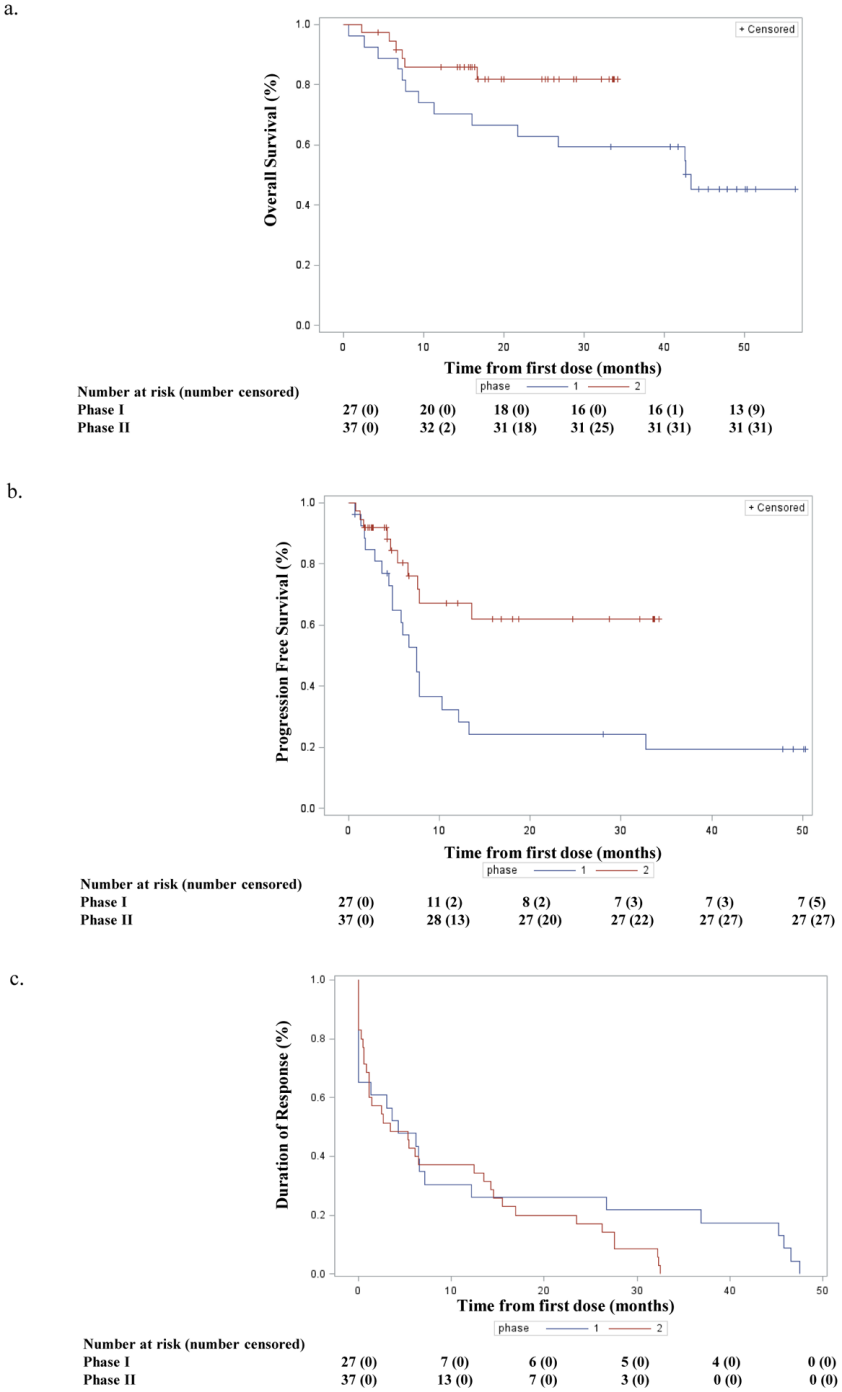
Overall Survival, Progression Free Survival and Duration of Response Time-to-event points for patients in the Phase 1 and 2 treated with BvB. In all cases, the red curve in each panel represents the results from the Phase 2.

The ORR in the combined Phase 1–2 portions was observed in forty-six of 65 (71%, 95% CI 58–81) patients, with 21 of 65 (32%) attaining a CR (Table 3). Despite being very heavily treated, 17 of 28 (ORR 61%, 95% CI 41–79) of patients achieved a response in the Phase 1, with 5 of 28 (18%) being CR. In the Phase 2, 29 of 37 (ORR 78%, 95% CI 62–91) patients responded, with 16 of 37 (43%) attaining a CR. Table 3 and Figure 3 present the response data as a function of the study phase. Figure 2 depicts responses based on percent change from tumor baseline as evaluated by functional imaging. Responses were seen in essentially every cohort, including 2 out of 6 patients attaining a CR at the lowest dose level of Bv (1.2mg/kg) and B (70mg/m2). Supplemental Tables 1 and 2 provide the responses for all patients on study, as well as their histology and extent of prior treatment (webappendix page 4–5). The mPFS among the phase 1 and 2 patients was 7.5 months (95% CI: 4.8 to 12.1), and not reached. In the Phase 2, the 1-year PFS was 67% (25 of 37 patients) and the 2-year PFS was 62% (23 of 37 patients). The median DOR in the Phase I was 4.3 months (95% CI 0 to 7.1) and 3.95 months (95% CI 7.5 to not reached) in the Phase 2, while the median OS respectively, was 43.3 months (95% CI 11.3 to not reached) and not reached. Moreover, in the Phase 2, the OS at 1- and 2-years was 86% (32 of 37 patients) and 82% (30 of 37 patients), respectively.

Since there is limited data on the serial analysis of HL biomarkers in this setting, extensive correlative studies were performed for all patients in the Phase 1 and 2. Statistical analyses exploring a correlation with pretreatment level, changes in biomarker as a function of treatment, and correlation in both cases with clinical response (CR, PR, SD, POD, DOR, PFS) established only one statistically significant correlation. No correlation was found with the pretreatment level of TARC, galectin-1 and CD163 and outcome, nor with any change in biomarker and clinical outcome (webappendix pages 7–10). The baseline CD30 in CRs was significantly lower than in the non-responders (SD + POD) (*p*=0.02) while the percentage change in sCD30 from baseline was not significantly different in the two groups (*p*=0.58) (webappendix page 6).

## DISCUSSION

This was a very heavily treated patient population. Sixty-five percent of patients received some form of stem cell transplantation, with the median number of prior therapies being 4. Despite being heavily treated, no MTD was reached leading to a RP2D of Bv of 1.8mg/kg and B at 90mg/m2 administered every 3 weeks. The incidence of Grade 3 or 4 peripheral sensory neuropathy, neutropenia and thrombocytopenia in the Phase 2 were 0%, 35% and 0%, respectively, likely owing to the fact that these patients received a comparatively limited number of cycles of therapy (median number of cycles administered 5). In contrast, the incidence of Grade 3 or 4 neutropenia and thrombocytopenia in the pivotal Bv data in HL was 8%, 20% and not reported (i.e. < 10% all grade). The incidence of Grade 3 or 4 neutropenia and thrombocytopenia seen with B in patients with HL was 9% and 20% respectively. These data confirm that the combination did not produce more toxicity than the single agents alone.

The combination of BvB was highly active. The ORR for the entire study population was 71%, and 78% in the Phase 2 portion of this study. Importantly, nearly half (43%) of the HL patients in the Phase 2 attained a CR. These data compare favorably with that seen for a number of other conventional salvage regimens used prior to ASCT. While there are only non-randomized studies to compare the activity of BvB against, it is noteworthy to point out that ICE chemotherapy produces an ORR and CR of 89% and 26% respectively^5^. Other platinum containing regimens like GND and GDP have been reported to produce ORR and CR of 70% and 19%, and 69% and 17% respectively^13^. Recently, a multicenter Phase 2 study exploring BvB exclusively in patients who relapsed after a single line of chemotherapy reported a ORR and CR of 92.5% and 73.5% respectively^14^. These data suggest that BvB demonstrates the ability to achieve CR with less chemotherapy, equivalent or less chemotherapy related toxicity, and without requiring an inpatient hospitalization often required for most patients receiving ICE. In fact, if CR is recognized as one of the most critical end-points prior to definitive ASCT, then the CR rates for the available regimens are as follows: BvB (this Phase 2 only) is 43%; BvB is 73.5%^14^; ICE is 26%^5^; GND is 19%^7^; GDP is 17%^9^; DHAP is 21%^6^; IGEV is 53%^10^; and BeGEV is 73%^11^. Bv has also been evaluated in early phase combination studies with DHAP and ESHAP in the pre-ASCT setting; both combinations show feasibility and early signs of efficacy with a high proportion of patients achieving CR by FDG-PET and successful stem cell mobilization^15, 16^. Recognizing this is a non-randomized study and comes with the limitations of all single arm Phase 2 studies, collectively, these data strongly suggest that BvB markedly improves the CR rate over other conventional salvage regimens, which theoretically could translate into improved cure rates for patients with HL in the salvage setting if coupled to ASCT. Clearly, further studies, preferably randomized trials, are warranted to allow for more accurate cross-regimen efficacy comparisons.

In the standard treatment of HL, patients who fail upfront therapy with any of the traditional treatment programs (ABVD, BEACOPP) may be cured with salvage therapy consolidated by an ASCT^5, 6, 17–19^. The most important feature of patients likely to be cured following ASCT is their ability to achieve a PET negative response following salvage therapy, that is, a complete remission. Strategies that circumnavigate mechanisms of acquired drug resistance may offer a better chance of achieving CR, and thus, improving the curability of the disease with ASCT. The pivotal data leading to approval of Bv was largely based on an exceptionally high ORR with a meaningful CR rate. The major liability of Bv lies in the fact that the PFS for patients achieving PR was equivalent to patients achieving only SD. Therefore, combination strategies with Bv could create opportunities for its use earlier in the disease natural history.

Previous biomarker studies in HL have established that the Hodgkin Reed-Sternberg cell specific chemokine CCL17 (also known as Thymus and Activation Regulated Chemokine (TARC)) is specific marker for HL disease activity^20–24^. Likewise, the soluble form of Galectin-1 and the M2 macrophage marker soluble CD163 have been found to be elevated in HL patients and correlate with disease progression^25–27^. Soluble CD30 has also been shown to be prognostic in HL^26, 28–30^. An extensive biomarker discovery initiative built into this trial revealed only a single statistically significant correlation between any marker and an outcome parameter. Baseline CD30 identified patients likely to achieve CR versus those unlikely to respond. It should be appreciated that of the available studies exploring the relationship between these biomarkers and a clinical correlate published to date, most have focused on patients with 0 to 1 prior therapy. For example, the pretreatment TARC in two studies where patients had only 1 line of prior therapy was 1856 pg/mL and 8,250 pg/mL respectively^21, 22^. In contrast, the median pretreatment TARC in this study was 16,634 pg/mL, and in a study of untreated patients was 28,013 pg/mL^20^. In the former studies, patients were comparatively less heavily treated, and there may be an enrichment for a more ‘favorable’ population. It is likely that the heavily treated nature of this study population is enriched for highly unfavorable patients, which may make these biomarkers less predictive in this setting.

We conclude BvB is very well tolerated even in highly treated patients, including those post ASCT and AlloSCT. In addition, the toxicity profile appears no worse compared to that seen with the single agents, and is administered in the outpatient setting. Second, it confirms that there is substantial clinical benefit, with an ORR and CR rate of 78% and 43% among patients with multiply relapsed HL. The responses are durable, with several more than 2 years in remission despite having relapsed post-transplant. Finally, it establishes that traditional biomarkers which may predict clinical outcomes in patients with a single-line of prior therapy, may not retain the same predictive value in heavily treated patients owing to the likely enrichment of a more „unfavorable‟ population. These data support the merits of BvB in the second-line and beyond, and may represent a significant improvement over other traditional salvage regimens.

## Supplementary Material

dummy_label

## Figures and Tables

**Table 1.** Patient Demographic and Clinical Characteristics

Parameter	Phase I (n=28)	Phase II (n=37)
**Age, years**		
Median	38	34
Range	25–70	18–72
**Sex**		
Male	18 (64%)	23 (62%)
Female	10 (36%)	14 (38%)
**Ethnicity**		
White, non-Hispanic	18 (64%)	25 (68%)
White Hispanic	0 (0%)	1 (3%)
Black, non-Hispanic	3 (11%)	2 (5%)
Asian/Indian/Pacific Islander	1 (4%)	5 (14%)
Other	6 (21%)	4 (11%)
**Disease**		
Hodgkin’s lymphoma	27 (96%)	37 (100%)
ALCL	1 (4%)	0 (0%)
**Prior Therapy**		
Median: 4 (range: 1–12)	5 (2–12)	3 (1–8)
ABVD/BEACOPP/EVA	27 (96%)	37 (100%)
Platinum based (ICE/ESHAP/DHAP)	28 (100%)	29 (78%)
Autologous stem cell transplant	21 (75%)	21 (27%)
Allogeneic transplant	2 (7%)	1 (3%)
Radiotherapy	12 (43%)	17 (46%)
Alkylator based (Lomustine/MOPP)	15 (54%)	2 (5%)
Lenalidomide	9 (32%)	3 (8%)
Vinblastine	10 (36%)	2 (5%)
Gemcitabine based (GCD, Gem/Vin)	9 (32%)	1 (3%)
Brentuximab vedotin	8 (29%)	3 (8%)
HDAC inhibitor	5 (18%)	0 (0%)
Experimental drug^1^	4 (14%)	3 (8%)
Etoposide	2 (7%)	0 (0%)

1 Experimental drugs included: HCD122 Lucatumumab (n=3), NAE Inhibitor MLN4924 (n=1), TGR-1202 (n=1), Azacitidine/Romidepsin (n=1), ACY1215 (n=1).

**Table 2.** Hematologic and Non-hematologic Toxicities That Occurred in ≥ 10% of the Study Population

	Phase I (n=28)					Phase II (n=37)				
	All	Grade 1	Grade 2	Grade 3	Grade 4	All	Grade 1	Grade 2	Grade 3	Grade 4
MedDRA Toxicity	No. (%)	No. (%)	No. (%)	No. (%)	No. (%)	No. (%)	No. (%)	No. (%)	No. (%)	No. (%)
Abdominal pain	4 (14%)	4 (14%)	0 (0%)	0 (0%)	0 (0%)	4 (11%)	0 (0%)	4 (11%)	0 (0%)	0 (0%)
Anemia	5 (18%)	0 (0%)	0 (0%)	5 (18%)	0 (0%)	2 (5%)	0 (0%)	2 (5%)	0 (0%)	0 (0%)
Anxiety	3 (11%)	2 (7%)	1 (4%)	0 (0%)	0 (0%)	1 (3%)	1 (3%)	0 (0%)	0 (0%)	0 (0%)
Back pain	3 (11%)	3 (11%)	0 (0%)	0 (0%)	0 (0%)	2 (5%)	0 (0%)	2 (5%)	0 (0%)	0 (0%)
Chills	6 (21%)	6 (21%)	0 (0%)	0 (0%)	0 (0%)	3 (8%)	3 (8%)	0 (0%)	0 (0%)	0 (0%)
Constipation	7 (25%)	4 (14%)	3 (11%)	0 (0%)	0 (0%)	7 (19%)	6 (16%)	1 (3%)	0 (0%)	0 (0%)
Cough	8 (29%)	4 (14%)	4 (14%)	0 (0%)	0 (0%)	6 (16%)	6 (16%)	0 (0%)	0 (0%)	0 (0%)
Diarrhea	8 (29%)	6 (21%)	2 (7%)	0 (0%)	0 (0%)	14 (38%)	10 (27%)	4 (11%)	0 (0%)	0 (0%)
Dysguesia	5 (18%)	5 (18%)	0 (0%)	0 (0%)	0 (0%)	4 (11%)	4 (11%)	0 (0%)	0 (0%)	0 (0%)
Dyspepsia	4 (14%)	4 (14%)	0 (0%)	0 (0%)	0 (0%)	4 (11%)	4 (11%)	0 (0%)	0 (0%)	0 (0%)
Dyspnea	10 (36%)	5 (18%)	5 (18%)	0 (0%)	0 (0%)	8 (22%)	8 (22%)	0 (0%)	0 (0%)	0 (0%)
Fatigue	15 (54%)	5 (18%)	10 (36%)	0 (0%)	0 (0%)	25 (68%)	10 (27%)	15 (41%)	0 (0%)	0 (0%)
Fever	7 (25%)	5 (18%)	2 (7%)	0 (0%)	0 (0%)	15 (41%)	5 (14%)	10 (27%)	0 (0%)	0 (0%)
Headache	4 (14%)	4 (14%)	0 (0%)	0 (0%)	0 (0%)	7 (19%)	7 (19%)	0 (0%)	0 (0%)	0 (0%)
Hot flashes	3 (11%)	3 (11%)	0 (0%)	0 (0%)	0 (0%)	0 (0%)	0 (0%)	0 (0%)	0 (0%)	0 (0%)
Hyperhidrosis	3 (11%)	3 (11%)	0 (0%)	0 (0%)	0 (0%)	0 (0%)	0 (0%)	0 (0%)	0 (0%)	0 (0%)
Infusion-related reaction	3 (11%)	1 (4%)	0 (0%)	2 (7%)	0 (0%)	4 (11%)	0 (0%)	4 (11%)	0 (0%)	0 (0%)
Lung infection	0 (0%)	0 (0%)	0 (0%)	0 (0%)	0 (0%)	5 (14%)	0 (0%)	0 (0%)	5 (14%)	0 (0%)
Myalagia	0 (0%)	0 (0%)	0 (0%)	0 (0%)	0 (0%)	2 (5%)	2 (5%)	0 (0%)	0 (0%)	0 (0%)
Nasal congestion	3 (11%)	3 (11%)	0 (0%)	0 (0%)	0 (0%)	2 (5%)	2 (5%)	0 (0%)	0 (0%)	0 (0%)
Nausea	15 (54%)	5 (18%)	10 (36%)	0 (0%)	0 (0%)	25 (68%)	20 (54%)	5 (14%)	0 (0%)	0 (0%)
Neutrophil count decreased	3 (11%)	0 (0%)	0 (0%)	3 (11%)	0 (0%)	13 (35%)	0 (0%)	0 (0%)	10 (27%)	3 (8%)
Pain	6 (21%)	6 (21%)	0 (0%)	0 (0%)	0 (0%)	6 (16%)	6 (16%)	0 (0%)	0 (0%)	0 (0%)
Peripheral sensory neuropathy	9 (32%)	0 (0%)	9 (32%)	0 (0%)	0 (0%)	2 (5%)	0 (0%)	2 (5%)	0 (0%)	0 (0%)
Platelet count decreased	4 (14%)	0 (0%)	0 (0%)	4 (14%)	0 (0%)	0 (0%)	0 (0%)	0 (0%)	0 (0%)	0 (0%)
Pruritis	8 (29%)	4 (14%)	4 (14%)	0 (0%)	0 (0%)	6 (16%)	0 (0%)	6 (16%)	0 (0%)	0 (0%)
Rash maculopapular	3 (11%)	3 (11%)	0 (0%)	0 (0%)	0 (0%)	12 (32%)	6 (16%)	6 (16%)	0 (0%)	0 (0%)
Sore throat	3 (11%)	3 (11%)	0 (0%)	0 (0%)	0 (0%)	0 (0%)	0 (0%)	0 (0%)	0 (0%)	0 (0%)
Vertigo	3 (11%)	3 (11%)	0 (0%)	0 (0%)	0 (0%)	3 (8%)	0 (0%)	0 (0%)	0 (0%)	0 (0%)
Vomiting	10 (36%)	2 (7%)	8 (29%)	0 (0%)	0 (0%)	12 (32%)	5 (14%)	7 (18%)	0 (0%)	0 (0%)

* Serious Adverse Event reported in ≥ 10% of patients included Fever (n=10).

** No grade 5 events were reported

**Table 3.** Summary of Response in Phase 1 and Phase 2

Response Type	HL/ALCL	HL	Total Population
	Phase I (n=27/1)	Phase II (n=37)	Phase I/II (n=65)
ORR	17 (61%, 41–79)	29 (78%, 62–91)	46 (71%, 58–81)
CR	5 (18%)	16 (43%)	21 (32%)
PR^1^	12 (43%)	13 (35%)	25 (38%)
SD	4 (14%)	5 (14%)	9 (14%)
NE	1 (4%)	0 (0%)	1 (2%)
POD	6 (21%)	3 (8%)	9 (14%)

Data provided are n (%, 95% CI) or n (%)

Abbreviations: CR, complete response; PR, partial response; SD, stable disease; NE, not evaluable; POD, progression of disease

1 ALCL subject included in PR.

## Panel: Research in Context

### Evidence before this study

To date, there are many studies exploring the optimal salvage regimen for patients with relapse or refractory classical Hodgkin lymphoma, with no one universally agreed upon standard of care, as all studies in this setting are non-randomized. We searched PubMed (in June 2012) for clinical studies (no restrictions on language or time) on this combination and did not find any reporting on the regimen of BvB. We used search terms of “brentuximab vedotin” and “bendamustine” with no papers identified. The development of the anti-CD30 antibody drug conjugate Bv has created an opportunity to explore the integration of this agent with other therapeutic drugs used in CD30 positive malignancies to improve outcome, and lighten the burden of conventional chemotherapy. In HL, Bv produces a high ORR, though the median PFS for the population is only 5.6 months, 21.7 months for patients in CR^1^. Patients achieving a PR or less do not have a PFS advantage compared to the entire study population. Likewise, bendamustine (B) produces an ORR of 53% in patients with relapsed or refractory HL, with a short median PFS of 5.2 months. Given the activity of both agents in HL and T-cell lymphoma, the combination was explored in order to improve the clinical benefit of these single agents.

### Added value of this study

Salvage regimens for patients with relapsed HL or T-cell lymphoma typically employ platinum based chemotherapy regimens. These regimens carry substantial toxicity, and exhibit poor CR rates in general. This combination of Bv and Bendamustine (BvB) integrates a recently approved biological agent, and offers the prospect of reducing reliance on platinum-based regimens in the salvage setting, leading to improved safety. The improvement in the CR rate, probably the single most prognostic determinant going into autologous stem cell transplant (ASCT), represents a major advance for this salvage regimen, and could lead to improved curative outcomes for these patients when coupled to stem cell transplant. This regimen, in a heavily treated patient population produced a high ORR and CR rate, enabling some patients to be successfully bridged to ASCT, with many individual patients experiencing extended duration time to event benefits.

### Implications of the available study

The marked activity of this regimen, including in heavily treated patients, with a high CR rate, offers the prospect that this salvage regimen could replace platinum-based chemotherapy regimens prior to autologous stem cell transplant. In addition, the favorable toxicity profile, coupled with it being an outpatient regimen, reduces the toxicity burden for these patients who have a high prospect of cure, hopefully reducing the long term complications of chemotherapy in this patient population. The immediate implications of the BvB regimen is that some patients can be bridged to ASCT, or experience durable time to event benefit despite having chemotherapy resistant disease.
