# Supplementary material for: BRENTUXIMAB VEDOTIN AND BENDAMUSTINE (BvB) IN PATIENTS WITH RELAPSED OR REFRACTORY HODGKIN LYMPHOMA: AN INTERNATIONAL, MULTICENTER, SINGLE-ARM, PHASE 1–2 TRIAL
Source: Lancet Oncol. Author manuscript; Available in PMC 2022 May 12. (PMC9098158; doi:10.1016/S1470-2045(17)30912-9)
Supplement: dummy_label [file NIHMS931226-supplement-dummy_label.pdf]

Supplementary Appendix

This appendix has been provided by the authors to give readers additional information about their work. Supplement to: O'Connor OA, Lue JK, Sawas A, et al. Brentuximab Vedoitn and Bendamustine (BvB) in Patients with Relapsed or Refractory Hodgkin Lymphoma: An International Multicenter, Single-Arm, Phase 1-2 Trial.

`

Table of Contents

Study Site and Investigators …………………………………………………………………………………….. 3

Supplementary Table 1 Detailed Patient Accounting and Dose Cohort Assignment from Phase I …………... 4

Supplementary Table 2 Detailed Patient Accounting and Dose Cohort Assignment from Phase II ………….. 5

Supplementary Figure 1 Baseline CD30 Predicts Clinical Response to BvB …………………………………… 6

Supplementary Figure 2 Serum TARC Levels Do Not Predict Clinical Response ……………………………... 7

Supplementary Figure 3 Serum CD163 Does Not Correlate with Clinical Response …………………………... 8

Supplementary Figure 4 Galectin Does Not Serve as a Biomarker for Clinical Response ……………………... 9

Supplementary Figure 5 Baseline TARC, CD163, and Galectin and Number of Prior Therapies …………... 10

Clinical Trial Protocol ………………………………………………………………………………………... 11

`

Study Sites and Investigators

The following investigators and institutions recruited patients for this study: John Kurvuilla —Princess Margaret Cancer Centre, Toronto, ON, Canada (29); Owen A. O'Connor—Center for Lymphoid Malignancies, Department of Medicine Columbia University Medical Center – College of Physicians and Surgeons, New York, N.Y (20); and Joseph M. Connors—BC Cancer Agency, Centre for Lymphoid Cancer Vancouver, BC, Canada (16).

`

***Supplemental Table 1*. Detailed Patient Accounting and Dose Cohort Assignment from Phase I**

Cohort	Patient ID	Prior Therapies	Number of Cycles (median: 6)	Toxicities	Best Response
**Brentuximab vedotin 1.2mg/kg Bendamustine 70mg/m2**	CU-001	11 (ASCT)	6	None	PR*
	CU-002	10 (ASCT)	1	None	POD*
	CU-003	7 ASCT)	6	No DLT	CR*
	CU-004	13 (ALLO, ASCT)	6	DLT - (diffuse rash)	CR
	CU-005	14 (ALLO, ASCT)	2	None	POD*
	BC-001	6 (ASCT)	6	None	PR*
	PM-001	6 (ASCT)	6	None	SD*
**Brentuximab vedotin 1.2 mg/kg Bendamustine 80 mg/m2**	PM-002	6 (ASCT)	6	None	PR*
	PM-003	6 (ASCT)	6	None	PR
	BC-002[1]	2	5	None	PR*
**Brentuximab vedotin 1.8 mg/kg Bendamustine 80 mg/m2**	CU-007	5	6	None	SD*
	CU-008	5 (ASCT)	6	None	PR
	PM-004	3 (ASCT)	6	None	PR
	PM-006	4 (ASCT)	6	None	CR
	BC-003	3	6	None	CR
	BC-004	8	4	None	CR
	BC-005	9	2	DLT - (Grade 4 Neutropenia)	POD*
**Brentuximab vedotin 1.8 mg/kg Bendamustine 90 mg/m2**	CU-009	6 (ASCT)	2	None	POD
	CU-010	4 (ASCT)	2	None	PR
	PM-007	11 (ASCT)	6	None	NE
	PM-008	3 (ASCT)	6	None	SD*
	PM-009	5 (ASCT)	6	None	SD
	PM-010	4 (ASCT)	5	None	PR
	PM-011	8 (ASCT)	2	None	PR*
	BC-006	8 (ASCT)	3	DLT - (Grade 4 Neutropenia)	POD*
	BC-007	4	3	None	POD*
	BC-008	4 (ASCT)	6	None	PR*
	BC-009	3	5	None	PR*

Abbreviations: ASCT, autologous stem cell transplant; ALLO, allogeneic stem cell transplant; DLT, dose-limiting toxicity; CR, complete response; NE, not evaluable; PD, progressive disease; PR, partial response; SD, stable disease.

[1]All subjects have HL except BC-002 who had ALCL.

*Denotes Death secondary to Progression of Disease

`

***Supplemental Table 2*. Detailed Patient Accounting and Dose Cohort Assignment from Phase II**

Cohort	Patient ID	Prior Therapies	Number of Cycles (median: 5)	Best Response
**Brentuximab vedotin 1.8 mg/kg Bendamustine 90 mg/m2**	CU-011	5 (ASCT)	6	CR
	CU-012	1	6	SD
	CU-013	9 (ALLO, ASCT)	6	SD*
	CU-014	1	5	CR
	CU-015	5 (ASCT)	6	CR
	CU-019	1	3	SD
	CU-020	1	4	SD
	CU-021	1	3	CR
	CU-022	2	6	PR
	CU-023	1	3	CR
	CU-024	6 (ASCT)	6	PR
	PM-012	6 (ASCT)	6	PR
	PM-013	3 (ASCT)	6	CR
	PM-014	4 (ASCT)	6	CR
	PM-015	4 (ASCT)	6	PR
	PM-016	3 (ASCT)	5	CR
	PM-017	7 (ASCT)	6	PR
	PM-018	4 (ASCT)	1	POD*
	PM-019	4 (ASCT)	6	PR**
	PM-020	4 (ASCT)	2	CR
	PM-021	3	2	PR
	PM-022	2	4	CR**
	PM-023	4 (ASCT)	6	CR
	PM-024	4 (ASCT)	2	POD
	PM-025	2	4	PR
	PM-026	4 (ASCT)	6	PR
	PM-027	2	5	PR
	PM-028	2	4	PR
	PM-029	3	2	CR
	PM-030	2	2	CR
	BC-011	1	2	POD*
	BC-012	5 (ASCT)	5	CR*
	BC-013	3 (ASCT)	3	CR
	BC-014	3 (ASCT)	2	CR
	BC-015	3 (ASCT)	5	CR
	BC-016	5 (ASCT)	6	SD*
	BC-017	3	3	CR

*Denotes Death secondary to Progression of Disease
**Denotes Death after transplant (autologous or allogeneic)
***All patients had a diagnosis of Hodgkin Lymphoma

`

Supplemental Figure 1. **Baseline CD30 Predicts Clinical Response to BvB**

a.

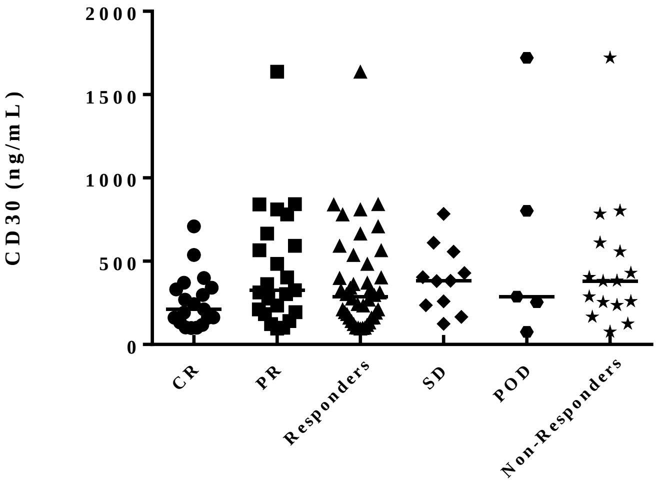

Pre-Treatment CD30	p-value
CR vs POD	0.33
CR vs SD	0.01
CR vs PR	0.06
Responders vs Non-Responders	0.14
CR vs Non-Responders	0.02

b.

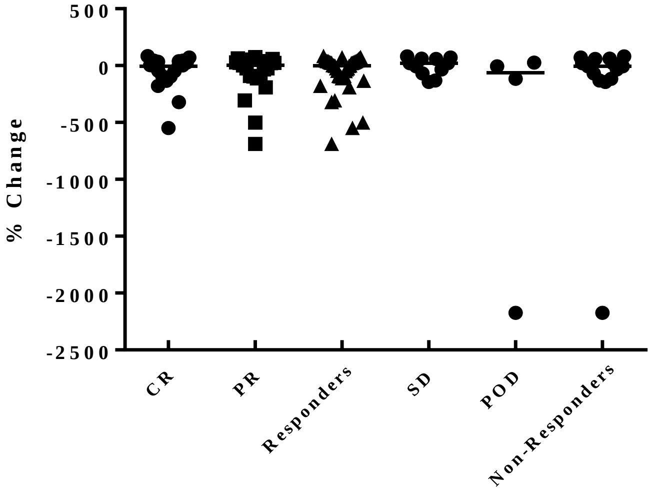

% Change in CD30	p-value
CR vs POD	0.40
CR vs SD	0.43
CR vs PR	0.30
Responders vs Non-Responders	0.58

* Baseline Serum Median (IQR) Values. Complete Response (CR): 200.44 (222.59); Partial Response (PR):311.79 (382.57); Stable Disease (SD):416.40 (351.41); Progression of Disease (POD): 270.69 (702.37); Non-Responders: 393.29 (393.46); Responders: 276.15 (238.70)

**Percent Change Median (IQR) Values. Complete Response (CR): 0.02 (1.32); Partial Response (PR): -0.02 (1.26); Stable Disease (SD): -0.21 (1.30); Progression of Disease (POD): 0.61 (11.55); Non-Responders: 0.06 (1.72); Responders: 0 (1.26)

Supplemental Figure 2. **Serum TARC Levels Do Not Predict Clinical Response**

a. b.

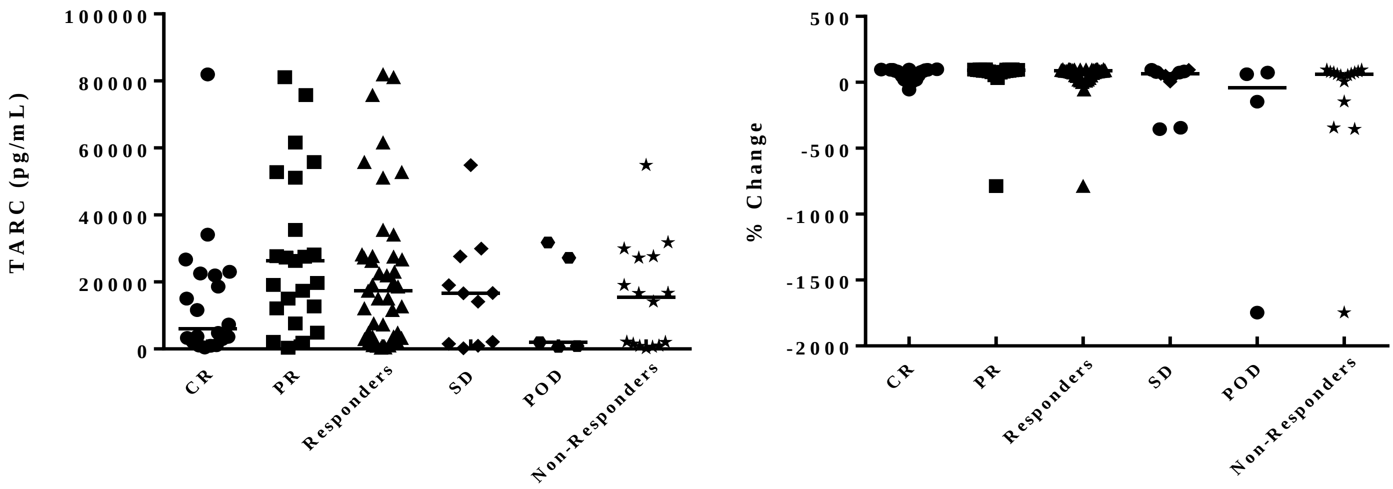

Pre-Treatment TARC	p-value
CR vs POD	0.59
CR vs SD	0.73
CR vs PR	0.17
Responders vs Non-Responders	0.49

% Change in TARC	p-value
CR vs POD	0.34
CR vs SD	0.25
CR vs PR	0.69
Responders vs Non-Responders	0.19

* Baseline Serum Median (IQR) Values. Complete Response (CR): 9459.40 (20454.92); Partial Response (PR):19151.20 (30665.60); Stable Disease (SD): 16656.83 (25595.91); Progression of Disease (POD): 2050.00 (26279.48); Non-Responders: 15370.65 (25872.11); Responders: 15007.80 (23653.88)

**Percent Change Median (IQR) Values. Complete Response (CR): -0.81 (0.75); Partial Response (PR): -0.87 (0.22); Stable Disease (SD): -0.74 (0.34); Progression of Disease (POD): 0.43 (10.15); Non-Responders: -0.63 (0.77); Responders: -0.86 (0.43)

`

***Supplemental Figure 3.* Serum CD163 Does Not Correlate with Clinical Response**

a.

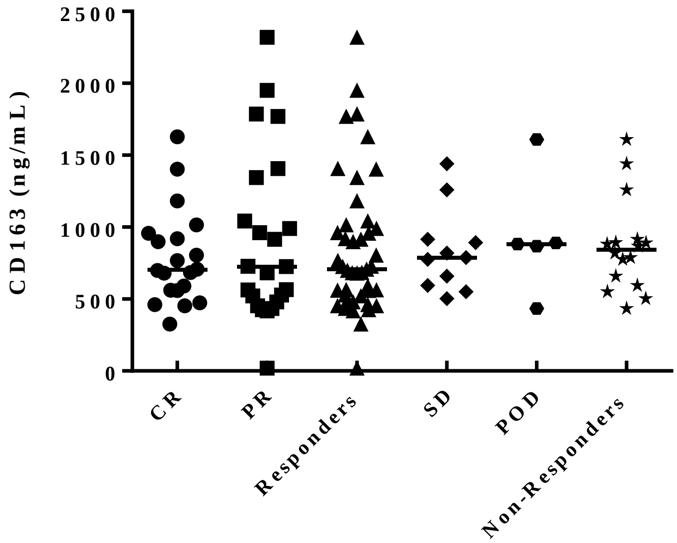

b.

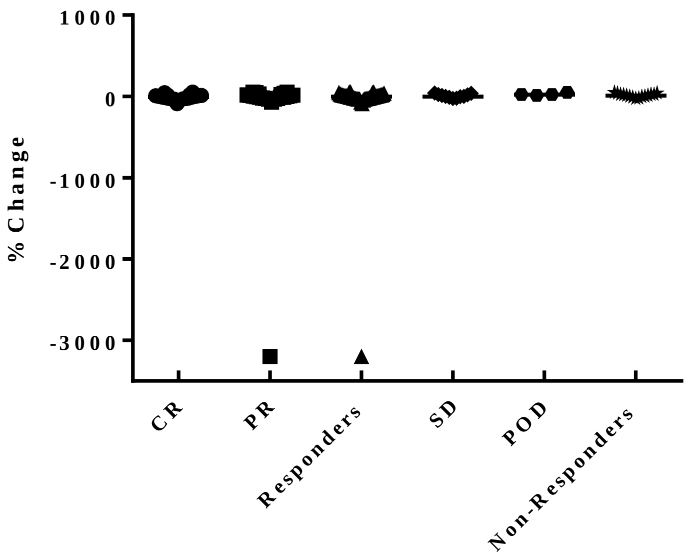

Pre-Treatment CD163	p-value
CR vs POD	0.96
CR vs SD	0.32
CR vs PR	0.87
Responders vs Non-Responders	0.54

% Change in Galectin	p-value
CR vs POD	0.07
CR vs SD	0.27
CR vs PR	0.94
Responders vs Non-Responders	0.12

* Baseline Serum Median (IQR) Values. Complete Response (CR): 702.69 (395.92); Partial Response (PR): 681.34 (516.30); Stable Disease (SD): 804.16 (601.07); Progression of Disease (POD): 875.14 (457.88); Non-Responders: 843.53 (461.43); Responders: 698.37 (438.47)

**Percent Change Median (IQR) Values. Complete Response (CR): 0.02 (0.34); Partial Response (PR): 0.04 (0.45); Stable Disease (SD): -0.06 (0.25); Progression of Disease (POD): -0.23 (0.20); Non-Responders: -0.11 (0.23); Responders: 0.04 (0.36)

`

Supplemental Figure 4. **Galectin Does Not Serve as a Biomarker for Clinical Response**

a.

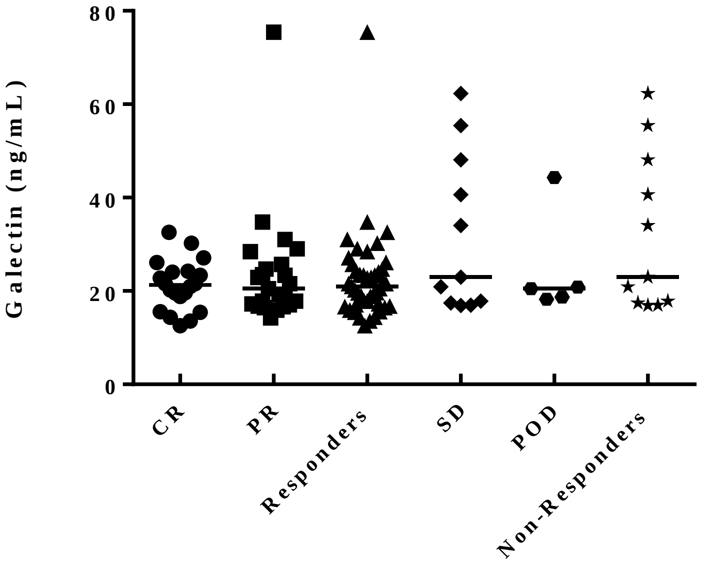

b

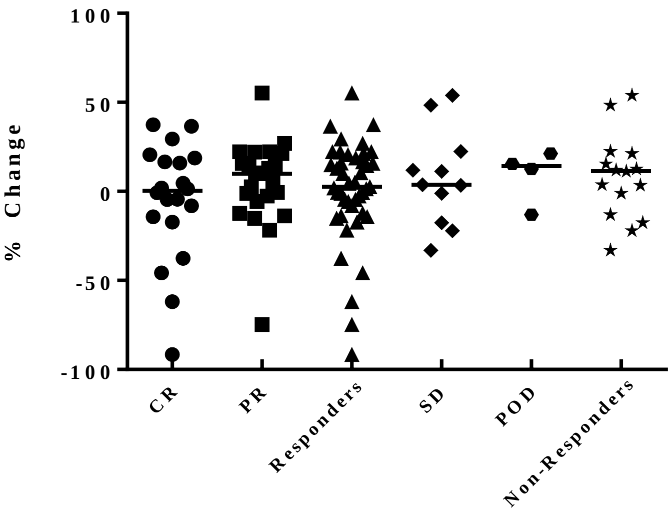

Pre-Treatment Galectin	p-value
CR vs POD	0.31
CR vs SD	0.10
CR vs PR	0.53
Responders vs Non-Responders	0.10

% Change in Galectin	p-value
CR vs POD	0.57
CR vs SD	0.42
CR vs PR	0.84
Responders vs Non-Responders	0.26

* Baseline Serum Median (IQR) Values. Complete Response (CR): 21.55 (5.34); Partial Response (PR): 20.49 (7.95); Stable Disease (SD): 21.92 (23.20); Progression of Disease (POD): 20.65 (22.32); Non-Responders: 20.84 (22.78); Responders: 20.99 (7.15)

**Percent Change Median (IQR) Values. Complete Response (CR): -0.02 (0.33); Partial Response (PR): -0.03 (0.28); Stable Disease (SD): -0.11 (0.23); Progression of Disease (POD): -0.14 (0.18); Non-Responders: -0.12 (0.22); Responders: -0.02 (0.29)

`

Supplemental Figure 5. **Baseline TARC, CD163, and Galectin and Number of Prior Therapies**

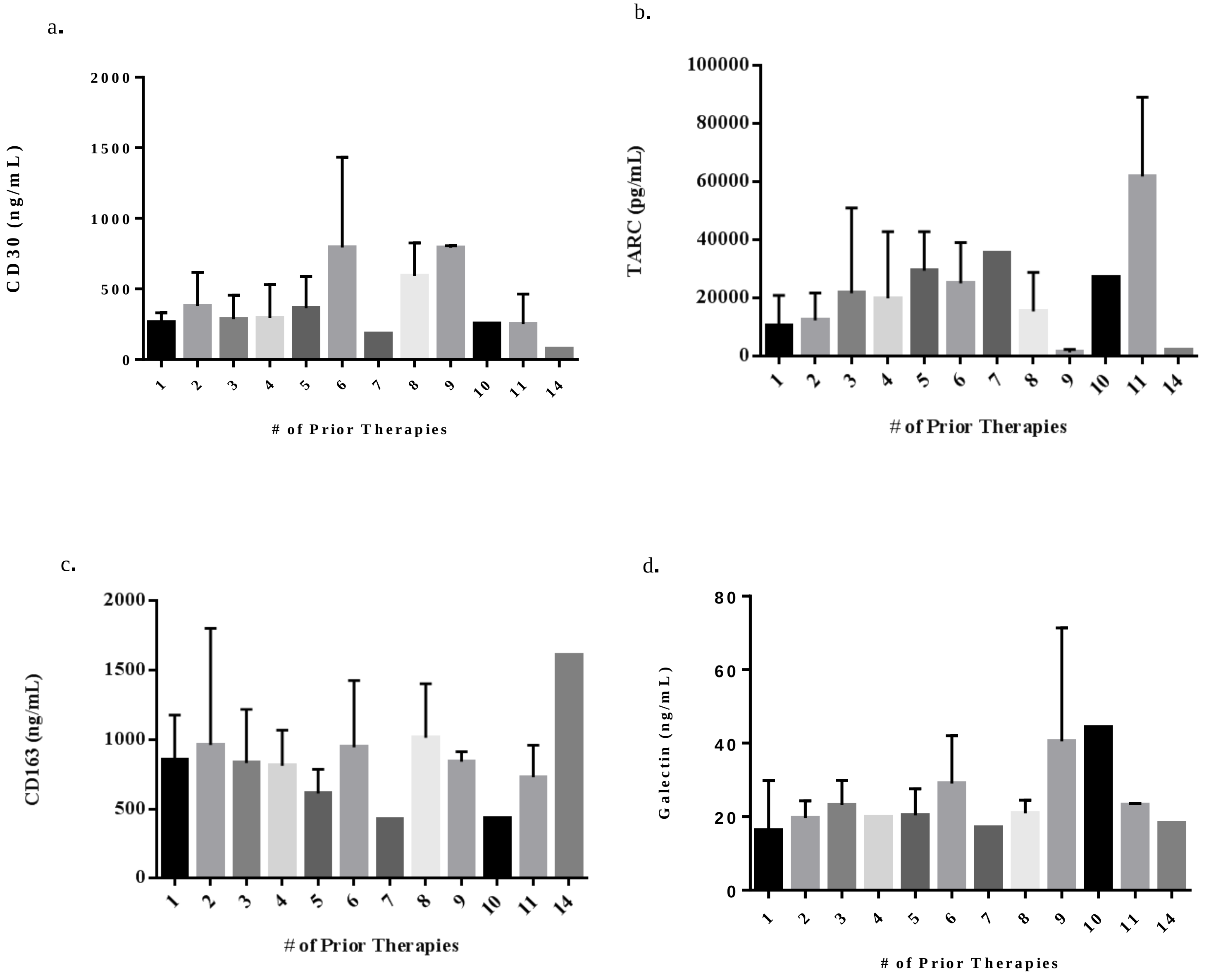

	R^2	Pearson's Correlation
**CD30**	2.00E-05	-0.06
**TARC**	0.1266	0.28
**CD163**	0.1143	0.44
**Galectin**	0.1672	0.35

A Phase I/II Clinical Trial of the Combination of Brentuximab Vedotin and Bendamustine in Patients with Relapsed or Refractory Hodgkin Lymphoma or Anaplastic Large Cell Lymphoma

Coordinating Center: Columbia University Medical Center (CUMC)

Participating Centers: BCCA Center for Lymphoid Cancer, British Columbia
Princess Margaret Hospital, University of Toronto

Principal Investigator: Owen A. O'Connor, MD, Ph.D.
Professor of Medicine and Developmental Therapeutics,
Director, Center for Lymphoid Malignancies
OO2130@columbia.edu

Co-Investigators: Changchun Deng, MD, Ph.D.
Assistant Professor of Clinical Medicine
CD2448@columbia.edu

Jennifer Amengual, MD
Assistant Professor of Clinical Medicine
JEA2149@columbia.edu

Ahmed Sawas, MD
Instructor in Clinical Medicine
AS4386@columbia.edu

John Kuruvilla, MD
Site Principal Investigator
Princess Margaret Hospital
Toronto, ON
John.Kuruvilla@uhn.on.ca

Joseph M. Connors, MD
Site Principal Investigator
Clinical Director, BC Cancer Agency Centre for Lymphoid Cancer
600 West 10th Avenue
Vancouver, BC V5Z 4E6 Canada
jconnors@bccancer.bc.ca

IND#: *115338*

CUMC Protocol #:
AAAJ5050

`

Protocol Type / Version # / Version Date: _*Amendment 2 / Version 2 / 22December2015*

PROTOCOL SUMMARY

Title

A Phase I/II Clinical Trial of the Combination of Brentuximab Vedotin and Bendamustine in Patients with Relapsed or Refractory Hodgkin Lymphoma or Anaplastic Large Cell Lymphoma

Primary Objectives: Phase 1

- To determine the maximum tolerated dose (MTD) of brentuximab vedotin and bendamustine
- To determine the dose limiting toxicities (DLT) of brentuximab vedotin and bendamustine

Secondary Objectives: Phase 1

- To evaluate the overall response rate (ORR) (complete response (CR) + partial response (PR)) for all patients
- To estimate the progression free survival (PFS) and duration of response (DOR) for all patients

Primary Objective: Phase 2

- To determine the ORR (CR + PR) for the combination of brentuximab vedotin and bendamustine

Secondary Objectives: Phase 2

- To confirm the safety and tolerability of the combination of brentuximab vedotin and bendamustine
- To evaluate the DOR, PFS and Overall Survival (OS) for the combination of brentuximab vedotin and bendamustine

Correlative Studies Phase 1 and Phase 2

- To evaluate serum Tarc levels in patients as a function of treatment with brentuximab vedotin and bendamustine
- To evaluate the level of peripheral blood lymphocyte expression of programmed death-1 (PD-1) as a function of treatment with brentuximab vedotin and bendamustine
- To evaluate the decline in serum levels of IL-10 and IL-6 as a function of treatment with brentuximab vedotin and bendamustine

`

Patient Population
Specific inclusion and exclusion criteria are outlined in section 4.1 and 4.2

Number of Patients
If all planned cohorts are treated, and the final dose level (dose level 5) is declared the MTD this study will treat 22 patients in the phase 1. If all planned cohorts are expanded in the phase 1 and the final cohort is reached, this study will treat up to 12 additional patients in the phase 1. If MTD is reached earlier, fewer than 22 patients will be treated at phase 1. The phase 2 component consists of a maximum of 37 patients. This study is estimated to treat 59 patients, and at maximum will treat 71 patients.

Study Design and Methodology
This is a phase 1/2 open label, multi-center study to assess the safety and efficacy of brentuximab vedotin in combination with bendamustine in patients with relapsed or refractory HL or anaplastic large cell lymphoma (ALCL). Dose escalation in phase 1 will proceed according to a standard 3 + 3 dose escalation design. There will be no intra-cohort dose escalation. Three patients will be assigned to the starting dose level 1. If no DLT is observed after one cycle of treatment, and if the start of cycle 2 treatment is not delayed greater than 7 days for any toxicity possibly related to drug, trial accrual proceeds to the next dose level and another cohort of 3 patients is enrolled. If 1 patient in a cohort experiences a DLT, then the cohort is expanded to 6 patients. If 2 of 3 or more patients in a cohort experience a DLT then the cohort will be designated the maximum administered dose (MAD) and the dose level below will be expanded. In no more than 2 of 6 patients experience a DLT, this dose level will be designated the MTD. If none of the additional patients experience a DLT (1 out of 6) then dose escalation continues. The MTD is defined as the highest dose level at which <33% of the dose cohort (0 of 3 or 1 of 6) experience a DLT in the first cycle of therapy. Up to a maximum of 10 patients will be added at the MTD to more fully characterize the safety of the drug combination. As long as <33% of patients experience a DLT, this dose cohort will remain the MTD.The phase 2 component will employ a 2-stage Simon design (optimum and minimax) that includes a first stage with 19 patients, and a total of 37 patients in the combined stages of the phase 2. With 19 patients in the first stage, the trial will be terminated if there are 12 or fewer responses after 6 cycles of therapy. If the trial proceeds to the second stage (> 12 responses in the first 19 patients) 18 additional patients will be enrolled for a total of 37 patients in the phase 2.

Treatments Administered
In this study, brentuximab vedotin will be administered as an outpatient IV infusion on day 1 of each 21-day cycle. Bendamustine will be given as an outpatient infusion on days 1 and 2 of a 21 day cycle. Patients may receive prophylactic pegfilgrastim on day 3 of each cycle, or filgrastim for 5 to 10 days, per investigator's discretion. Patients can receive a maximum of 6 cycles of therapy.

Table 1: Drug Administration Schema

`

Day	1	2	3	4-21
**Brentuximab Vedotin IV**	↓			**Rest**
**Bendamustine IV**	↓	↓		
**Neulasta SQ* (per investigators discretion after cycle 1)**			↓	

Following the first cycle of therapy disease status will be evaluated using standard criteria. The safety of brentuximab vedotin will be monitored throughout the trial via laboratory test and adverse event (AE) collection at routine visits. Antitumor activity will be assessed at the end of cycles 2 and 6. Response determination will be based on the Revised Response Criteria for Malignant Lymphoma (33).

Study Schema

Figure 1: Study Schema

Screening

History, Physical, Performance Status
Blood tests, PET/CT scan, Confirm pathology and CD30 expression

↓

CYCLE 1

Intervention

Brentuximab vedotin administered intravenously on day 1 of 21 day cycle
Bendamustine administered intravenously on days 1 and 2 of 21 day cycle

Evaluations

Safety evaluation on days: 1, 2, 8, 15
Laboratory evaluation days: 1, 2, 8, 15
Research blood draw days: 1, 8

↓

Future Cycles

Intervention

Brentuximab vedotin administered intravenously on day 1 of 21 day cycle
Bendamustine administered intravenously on days 1 and 2 of 21 day cycle

Evaluations

Safety evaluation on day 1 and 2 of each cycle
Laboratory evaluation day 1 of each cycle
Research blood , on day 1 of cycles 2 &4 and at EOT (within 14 days of last dose)
PET/CT (or CT) scan after cycles 2 & 6 (or last cycle of treatment), and thereafter every 3 to 6 months until disease recurrence or initiation of additional anti-lymphoma treatment

↓

Discontinuation

- Disease progression
- Intercurrent illness that prevents further administration of treatment
- Unacceptable adverse event(s), DLTs
- Pregnancy
- Patient decides to withdraw from the study
- General or specific changes in the patient's condition render the patient unacceptable for further treatment in the judgment of the investigator

`

LIST OF ABBREVIATIONS AND DEFINITIONS OF TERMS

ADC antibody drug conjugate
AE adverse event
ALCL anaplastic large cell lymphoma
ALT alanine aminotransferase
ANC absolute neutrophil count
ASCT autologous stem cell transplant
AST aspartate aminotransferase
ATA anti-therapeutic antibody
β -hCG beta human chorionic gonadotrophin
CBC complete blood count
CI confidence interval
CMV cytomegalovirus
CRF case report form
CR complete remission
CT computed tomography
CTCL cutaneous T-cell lymphoma
CTCAE Common Terminology Criteria for Adverse Events
DLBCL diffuse large B-cell lymphoma
DLT dose limiting toxicity
ECL electrochemiluminescence
ECOG Eastern Cooperative Oncology Group
EOT end of treatment
EE efficacy evaluable
ELISA enzyme-linked immunosorbent assay
GCP Good Clinical Practice
G-CSF granulocyte stimulating factor
GM-CSF granulocyte macrophage colony stimulating factor
HAART highly active antiretroviral therapy
HEENT head, eyes, ears, nose, and throat
HIPAA Health Information Portability and Accountability Act
HGBA1C hemoglobin A1C
HL hodgkin lymphoma
HRA Health Regulatory Agency
HRS hodgkin Reid-Sternberg cell
ICH International Conference on Harmonisation
IEC Independent Ethics Committee
IL-6 Interleukin 6
IL-10 Interleukin 10
IRB Institutional Review Board
IV intravenous
MCL mantle cell lymphoma
MedDRA Medical Dictionary for Regulatory Affairs
MMAE monomethylauristatin E
MTD maximum tolerated dose
NCI National Cancer Institute
NHL non-Hodgkin lymphoma
NYHA New York Heart Association
ORR overall objective response rate
OS overall survival
PCR polymerase chain reaction
PCP pneumocystis carinii (jiroveci) pneumonia

`

PD-1 programmed death -1
PDL-1 programmed death ligand-1
PET positron emission tomography
PFS progression-free survival
PK pharmacokinetic
PR partial remission
REB Research Ethics Board
SAE serious adverse event
SAP statistical analysis plan
SD stable disease
SMC Safety Monitoring Committee
SPD sum of the product of diameters
TEN toxic epidermal necrolysis
TIL tumor infiltrating lymphocyte
TH-2 T-helper-2
ULN upper limit of normal
USP United States Pharmacopeia

`

Table of Contents

PROTOCOL SUMMARY 12
Study Schema 15
LIST OF ABBREVIATIONS AND DEFINITIONS OF TERMS 16
1.0 Background 20
1.1 Background on Hodgkin Lymphoma 20
1.2 Background on Anaplastic Large Cell Lymphoma 20
1.3 Mechanism of Action of Brentuximab Vedotin 21
1.4 Preclinical Data for Brentuximab Vedotin 22
1.5 Clinical Safety and Efficacy of Brentuximab Vedotin 22
1.6 Mechanism of Action of Bendamustine 23
1.7 Clinical Safety and Efficacy of Bendamustine 23
1.8 Rationale for the Combination of Brentuximab Vedotin and Bendamustine 24
1.9 Background and Rationale for Correlative Studies 25
2.0 OBJECTIVES 26
2.1 Primary Objectives: Phase 1 26
2.2 Secondary Objectives: Phase 1 26
2.3 Primary Objective: Phase 2 26
2.4 Secondary Objectives: Phase 2 27
2.5 Correlative Studies Phase 1 and Phase 2 27
2.6 Endpoints 27
3.0 INVESTIGATIONAL PLAN 27
3.1 Summary of Study Design 27
3.2 Rationale for Selection of Doses and Treatment Schedule 30
4.0 STUDY POPULATION 31
4.1 Eligibility Criteria 31
4.2 Exclusion Criterion 32
4.3 Inclusion of Woman and Minorities 32
4.4 Removal of Patients from Therapy or Assessment 32
4.5 Criteria for Removal from Study 33
4.6 Early Discontinuation of Study Drug 33
4.7 Replacement of Withdrawn Patients 33
5.0 TREATMENT PLAN 33
5.1 Agent Administration and Schedule 33
5.2 Brentuximab Vedotin Administration 34
5.3 Bendamustine Administration 35
5.4 Required Premedications and Prophylaxis 36
5.5 Phase 1 Dose Escalation and Reduction Rules 37
5.6 Dose De-escalation Decision Rules 39
5.7 Dose-Limiting Toxicity 39
5.8 Dosing Delays and Dose Modifications 40
5.9 Concomitant Therapy 41
5.10 Treatment Compliance 41
5.11 Treatment Duration 42
6.0 STUDY ACTIVITIES 42
6.1 Study Procedures 42
6.2 Registration Procedures 42
6.3 Registration Process 43
6.4 Schedule of Events 44
6. 5 .... Required Blood Parameters and Other Investigations Prior to Each Treatment 45
6.6 .... End of Study Visit 45
`

7.0 STUDY ASSESMENTS 45
7.1 SCREENING/BASELINE ASSESSMENTS 45
7.2 SAFETY ASSESSMENTS 45
7.3 LYMPHOMA ASSESSMENTS 45
7.4 OTHER STUDY ASSESSMENTS 46
7.5 ASSESSMENT OF CORRELATIVE STUDIES 47
7.6 HANDLING OF SPECIMENS 47
8.0 ADVERSE EVENTS 48
8.1. DEFINITIONS 48
8.2 PROCEDURES FOR ELICITING AND RECORDING ADVERSE EVENTS 50
8.3 REPORTING PERIODS AND FOLLOW-UP FOR ADVERSE EVENTS AND SERIOUS ADVERSE EVENTS 52
8.4 IMMEDIATE REPORTING OF SERIOUS ADVERSE EVENTS 52
8.5 IND SAFETY REPORTS 53
9.0 DATA QUALITY CONTROL AND DATA ASSURANCE 53
9.1 MONITORING 53
9.2 DATA SAFETY MONITORING BOARD 53
10.0 DATA ANALYSIS METHODS 54
10.1 STUDY DESIGN 54
10.2 GENERAL STATISTICAL CONSIDERATIONS 55
10.3 PATIENT CHARACTERISTICS 56
10.4 ANALYSIS POPULATIONS 56
10.5 HANDLING OF MISSING DATA 56
10.6 SAFETY ANALYSIS 56
10.7 CLINICAL LABORATORY RESULTS 57
10.8 OTHER SAFETY ANALYSIS 57
10.9 EFFICACY ANALYSIS 57
10.10 ANALYSIS OF SECONDARY ENDPOINTS 57
10.11 ANALYSIS OF CORRELATIVE STUDIES 58
11.0 INFORMED CONSENT 58
11.1 STUDY INFORMED CONSENT 58
12.0 REFERENCES 60
13.0 APPENDICES 62

`

1.0 Background

The CD30 positive (CD30) lymphoproliferative malignancies typically include anaplastic large cell lymphoma and Hodgkin Lymphoma. In addition, it is estimated somewhere between 20-30% of B-Cell lymphomas, and mature T-Cell lymphomas also express CD30. We anticipate HL and ALCL will comprise the majority of our patient population, and thus the study will only be open to these histologies at this time.

1.1 Background on Hodgkin Lymphoma

Hodgkin lymphoma (HL) is a lymphoid neoplasm defined by the presence of malignant Hodgkin-Reed-Sternberg (HRS) cells against a background of inflammatory cells. Hodgkin lymphoma accounts for approximately 10 percent of all lymphomas and approximately 0.6 percent of all cancers diagnosed in the developed world annually (1). In 2009 8,500 of HL were diagnosed in the United States (2), and approximately 900 in Canada (3).

While HL is considered to be among one of the more curable lymphomas, 20% to 30% of patients will relapse after attaining remission, or fail to achieve a remission with first line chemotherapy (primary refractory disease). Only approximately 40% of these patients can be cured with current standard treatments which usually consists of second line chemotherapy and autologous stem cell transplantation (ASCT). For patients who relapse beyond second line therapy, or have primary refractory disease, treatment options are extremely limited (4-7). Improving the CR rate for these patients, may allow more of these young patients to proceed to successful autologous or allogeneic transplantation, currently the only potentially curative option for patients with multiply relapsed or refractory disease.

1.2 Background on Anaplastic Large Cell Lymphoma

Systemic anaplastic large cell lymphoma (sALCL) was first recognized by as a lymphoma characterized by large pleomorphic lymphoid cells expressing CD30 (8). In approximately 50% to 80% of cases, sALCL is associated with a t(2;5)(p23;q35) chromosome translocation relocating the anaplastic lymphoma kinase (ALK) gene on chromosome 2 to fuse with the NPM (nucleophosmin) gene on chromosome 5. In general, patients with ALK+ sALCL exhibit a superior overall survival and failure-free survival rate compared to ALK- sALCL (8).

Primary sALCL is a rare disease and accounts for 2% to 5% of all cases of adult non-Hodgkin lymphomas (NHL) (9, 10). It is estimated that approximately 66,120 new cases of NHL will be diagnosed in the United States in 2008 and 19,160 patients will die of their disease (11). It is thus estimated that 1,000 to 3,500 new cases of sALCL will be diagnosed in the United States in 2008 (11).

`

Approximately 40% to 65% of patients with sALCL develop recurrent
disease. Systemic ALCL is a rare malignancy, and is often included as a subset of aggressive lymphomas in large prospective studies of salvage therapy. Few effective therapies exist to salvage relapsed or refractory sALCL, both pre- and post-ASCT. A second complete remission (CR) with standard salvage chemotherapy such as EPOCH, ESHAP, or ICE is achieved in only 25% to 30% of patients, and the duration of second remission is frequently less than 1 year (12, 13). Novel treatments for these patients are desperately needed.

1.3 Mechanism of Action of Brentuximab Vedotin

Brentuximab vedotin is an antibody drug conjugate (ADC) directed against the CD30 antigen, and is being developed to treat patients with CD30-positive hematologic malignancies. The cell surface antigen CD30 is a member of the tumor necrosis receptor super-family. CD30 is expressed primarily on HL HRS cells and in ALCL, however CD30 expression has also been reported in: Kaposi's sarcoma (KS), cutaneous T-cell lymphomas (CTCL), mature T-Cell lymphomas a fraction of diffuse large B-cell lymphomas (DLBCL), some follicular lymphomas, and other lymphoproliferative diseases (14-17). CD30 in normal lymphocytes is expressed only by activated B-cells and T-cells, and for the latter CD30 is preferentially expressed by activated T cells producing T helper 2 (Th2) type B cell stimulatory cytokines.

Brentuximab vedotin consists of the chimeric antibody SGN-30 (cAC10) chemically conjugated to a synthetic analog (monomethylauristatin E [MMAE]) of the naturally occurring antitubulin agent dolastatin 10. Brentuximab vedotin is proposed to have a multistep mechanism of action that is initiated by binding to CD30 on the cell surface and internalization of the ADC. Upon trafficking to lysosomes, MMAE is released from the conjugate through proteolytic degradation of the drug linker (18). Binding of released MMAE to tubulin disrupts the microtubule network, leading to G2/M phase cell cycle arrest and apoptosis (19).

1.3.1 Chemical Structure of MMAE (the Drug Conjugate of Brentuximab Vedotin)

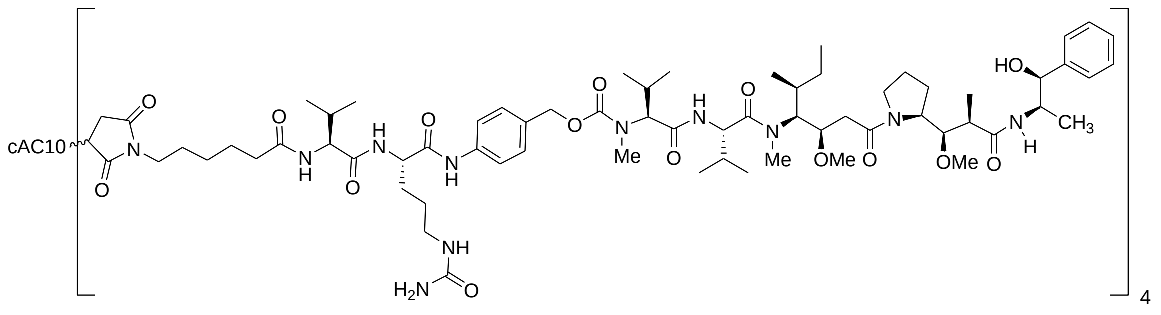

1.4 Preclinical Data for Brentuximab Vedotin

Preclinical and toxicologic studies of brentuximab vedotin demonstrated antitumor activity in both *in vitro* and *in vivo* models. The toxicity of multiple doses of brentuximab vedotin has been assessed in rats and monkeys. In both species, hypocellularity of the bone marrow and lymphoid depletion of the thymus were observed. Histopathologic lesions were also observed in the spleen in monkeys and in the liver and testes in rats. In addition, decreases in peripheral blood counts were observed in both species, and elevations in liver enzymes were seen in rats only. The most significant clinical toxicity was neutropenia, observed in monkeys, which resulted in secondary bacterial infections leading to early deaths at the 6 mg/kg dose. Toxicity was dose-dependent, with a no-observable-adverse-effect level of 0.5 mg/kg in rats and 1 mg/kg in monkeys. See the brentuximab vedotin Investigator's Brochure for details of the non-clinical data (Appendix pending).

1.5 Clinical Safety and Efficacy of Brentuximab Vedotin

A Phase 1, single-arm, open-label, dose-escalation study of brentuximab vedotin has been conducted in patients with CD30-positive hematologic malignancies. In this study, 42 evaluable patients received brentuximab vedotin at doses ranging from of 0.1 to 3.6 mg per kilogram of body weight on Day 1 of a 21-day cycle. Tumor response assessments were performed between the second and third cycle of therapy. Brentuximab vedotin was generally well-tolerated at doses of up to 1.8 mg/kg. The most common adverse events reported, primarily grade 1 or 2 in severity, were: fatigue (16 patients, 36%), pyrexia (15 patients, 33%), and diarrhea, nausea, neutropenia, and peripheral neuropathy (10 patients, 22% each). At the 1.2 mg/kg dose level (one level below the MTD) there were no grade 3 or 4 adverse events. Grade 3 neutropenia, back pain, and limb pain each occurred in 1 patient out of 12 (25%) at the 1.8 mg/kg dose level. There were no grade 4 adverse events at the 1.8mg/kg dose level. At the higher dose levels grade 3 neutropenia and pyrexia was seen in 2 of 12 (17%) patients who received the 2.7mg/kg dose, and grade 4 neutropenia and pyrexia in 1 of 1 (100%) patients receiving the 3.6 mg/kg dose (20).

Peripheral neuropathy was reported in 16 patients (36%), 13 of whom were treated at the 1.8-mg or 2.7-mg dose. Patients with peripheral neuropathy typically presented with grade 1 or 2 parasthesias in the hands or feet. Three patients discontinued treatment because of peripheral neuropathy. Resolution of peripheral neuropathy was noted in 10 of 16 patients (63%) at the last safety assessment for the study (20).

Objective responses were observed in 17 patients, including 13 complete remissions. Six of 12 (50%) of patients who received the maximum tolerated dose of 1.8mg/kg had an objective response. Tumor regression was noted in 36 of 42 (86%) of evaluable patients. Of 16 patients with disease-related symptoms at baseline (puritis, night sweats), 13 (81%) had resolution of their symptoms on therapy, irrespective of their anti-tumor response status (20).

The data from the phase II Pivotal trial in 102 patients with HL that had

`

relapsed after high dose chemotherapy and autologous stem cell transplant were reported at ASH 2010. This report noted an overall measurable tumor reduction in >90% of patients, a 75% ORR (CR + PR) and a 34% CR rate.

The efficacy and safety of brentuximab vedotin in patients with relapsed or refractory ALCL, at a dose of 1.8 mg/kg administered every 3 weeks for up to 16 cycles of treatment, has also been investigated. The spectrum of toxicity seen in patients with ALCL did not vary substantially from that seen in patients with HL.

Brentuximab vedotin was granted accelerated approval by the FDA in August 2011 for the treatment of relapsed or refractory HL in patients with progressive disease after autologous SCT, and for patients after 2 prior chemotherapy regimens, that are ineligible for transplantation. Provisional approval was also granted for patients with ALCL who have failed at least one prior treatment.

1.6 Mechanism of Action of Bendamustine

Bendamustine is a unique bifunctional mechlorethamine derivative conjoined to a purine-like benzamidazole ring. The exact mechanism of action of bendamustine is unknown, but mechlorethamines and their derivatives form electrophilic alkyl groups which form covalent bonds with electron-rich neutrophilic moieties, resulting in intrastrand DNA crosslinks. This bifunctional covalent linkage can lead to cell death via several pathways, and bendamustine is known to be active against both quiescent and dividing cells. The chemical structure of bendamustine is shown below.

1.6.1 Chemical Structure of Bendamustine

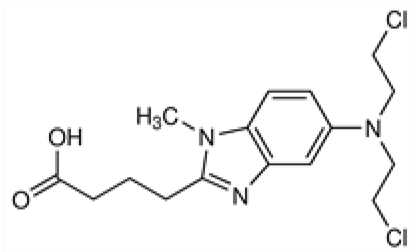

1.7 Clinical Safety and Efficacy of Bendamustine

Bendamustine has been in clinical use since the 1960s in the former East Germany. In the US bendamustine is FDA approved for the first line treatment of chronic lymphocytic leukemia (CLL) after a phase 3 randomized clinical trial comparing bendamustine at a dose of 100 mg/m2 on days 1 and 2 of a 28 day cycle to chlorambucil demonstrated superiority of bendamustine to chlorambucil, with ORR of 59% vs. 26% and a PFS of 18 months vs. 6 months in 301 CLL patients ($p < 0.0001$) (21).

Bendamustine has also demonstrated activity in two multi-center phase 2 studies of patients with relapsed and refractory indolent NHL or mantle cell lymphoma (MCL) (22, 23). In the first study 63 patients in first to third relapse or with refractory disease

`

received bendamustine at a dose of 90 mg/m2 on days 1 and 2 in combination with rituximab 375 mg/m2 every 4 weeks. Fifty-seven of 63 patients responded to bendamustine for an ORR of 90%. Median PFS was 24 months (range 4 to 44+ months). Myelosuppression was the major toxicity with 16% grade 3 and 4 leukopenia (23). A second phase 2 multi-center study of 67 patients with relapsed indolent NHL or MCL with documented rituximab resistance were treated with the same dose and schedule of bendamustine and rituximab for 4 to 6 cycles. The ORR was 92% with 41% CR, 14% unconfirmed CR, and 38% PR). Median DOR was 21 months (95% CI 18 to 24 months) and the median PFS was 23 months (95% CI 20 to 26 months). The primary toxicities were myelosupression (grade 3 or 4 neutropenia 36%, and grade 3 or 4 thrombocytopenia 9%) (22). In the front -line setting bendamustine in combination with rituximab has demonstrated activity in patients with advanced low grade NHL similar or superior to RCHOP (24).

The data in support of the activity of bendamustine in HL are growing. In 1975 a German study reported response in 7/10 untreated HL patients (25). Recently bendamustine produced a response in 12/14 patients with relapsed/refractory HL including 6 PR and 6 CR (26).

There are extensive safety data for bendamustine both alone and in combination with rituximab. The primary toxicity of bendamustine is myelosuppression and concomitant cnscns of infection. Infusion reactions and skin reactions to bendamustine have been observed. Tumor lysis has been observed in association with bendamustine in cycle 1 for patients with large tumor burdens. The use of allopurinol and bendamustine concurrently appears to increase the risk of dermatologic toxicity. Skin reactions have been reported when bendamustine has been given in combination with rituximab and include rash, pruritis, and bullous exanthema. Cases of Steven-Johnson syndrome and toxic epidermal necrolysis (TEN) have been reported when bendamustine was administered concurrently with allopurinol and/or rituximab.

1.8 Rationale for the Combination of Brentuximab Vedotin and Bendamustine

The curative treatment of relapsed or refractory lymphomas is not likely to be accomplished by the use of single agent therapies. The development of brentuximab vedotin is a promising therapeutic modality for patients with HL and other CD30 expressing hematologic malignancies, and has the potential to improve clinical outcome. Yet further treatment strategies are needed for the many patients who do not respond to brentuximab vedotin, or who relapse after brentuximab vedotin therapy.

The identification of rational combinations of novel therapies with disease specific activity has the potential to provide new clinical platforms for patients who have relapsed or are refractory to conventional chemotherapy. The rationale behind combination therapy is to employ agents with novel and complementary mechanisms of action which are rationally designed to target the key pathogenetic and pathognomonic features of specific diseases. The ideal therapeutic platform may be one of synergy between these novel agents, in which these targeted and conventional therapies have different spectrums of non-overlapping toxicities, and are rationally employed to maximize the sensitivity of the HL or ALCL to each other.

`

Combining brentuximab vedotin with a novel alkylating agent such as bendamustine could potentially improve the overall response rate, complete response rate, and response duration for relapsed/refractory HL and ALCL patients, with minimal additional toxicity. **This may be a valid strategy for remission induction as a bridge to autologous or allogeneic stem cell transplantation, in patients with relapsed or refractory HL and ALCL or a stand-alone secondary regimen for control of symptomatic disease and extension of survival.** Maximal cytoreduction prior to autologous or allogeneic stem cell transplant confers the greatest potential benefit from transplant. The current standard of care using second line chemotherapy regimens such as ICE (ifosfamide, cyclophosphamide, and etoposide) produce a ORR rate of 82% (reported in a single institution study) but a CR rate of only 26% (27). The CR rate of brentuximab vedotin as a single agent in Phase II is reported to be 34% while the bendamustine CR rate exceeds 30% (26). Underlying the rationale of this trial is the goal of identifying a novel synergistic combination strategy that will have the potential both for a high ORR and CR rate, which may move forward into an expanded trial as a treatment strategy for patients in first relapse prior to autologous transplantation, as well as patients with relapsed/ refractory HL as a bridge to allogeneic transplantation, or even as a stand-alone secondary regimen for control of symptomatic disease.

1.9 Background and Rationale for Correlative Studies

At present there are few correlative studies which appear applicable to the effects of these drugs, nor the underlying biology of these diseases. While some have been established in HL, the experience with these biomarkers in diseases other than HL are limited. Because we anticipate HL will be the predominant disease population enrolled, we will explore these markers in all patients with HL, recognizing they will be exploratory in all other subtypes of NHL.

1.9.1 Significance of Tarc in HL

The malignant Hodgkin lymphoma Reed-Sternberg cell (HRS cell) typically resides in a milieu of reactive inflammatory cells, comprising a very small fraction (0.1% to 10%) of the total cellular population. Growing data suggests that the inflammatory milieu surrounding the HL HRS cell has a vital role supporting the HL RS cells by secreting survival factors (28), and providing a permissive microenvironment for tumor cell growth. The HRS cells in turn secrete abundant amounts of the chemokine thymus and activation-regulated chemokine (TARC or CCL17), which preferentially attracts type 2 helper (Th2) cells and regulatory T cells (Tregs) to the tumor microenvironment, generating local immunosuppression. Serum Tarc levels are noted to be high in patients with active HL, and have been noted to decline in response to HDAC inhibition (29), and in small numbers of patients treated with brentuximab vedotin in a Phase 1 trial (20). The measurement of serum Tarc levels in a larger population of patients would provide further data in support and validation of this novel biomarker, and perhaps give us some clues as to the off target effects of brentuximab vedotin.

1.9.2 Significance of PD-1 and PDL-1 in HL

Programmed death-1(PD-1) and Programmed death-1 ligand (PD1-L) signalling are involved in the

`

functional impairment and "exhaustion" of T cells such as in chronic viral infection or tumor immune evasion. The interaction of PD-1 with its ligand PD-1L suppresses T cell function. Up-regulation of PD-L has been demonstrated both in HL cell lines, and in primary HRS cells (30). PD-1 is markedly elevated in both the tumor infiltrating lymphocytes and the peripheral T cells of HL patients (31), and likely plays a key role in inducing the permissive HL mileu. One component of the deficient anti-tumor immunity observed in HL patients may consequently be a function of T cell exhaustion induced by the activation of the PD-1-PD-L signalling pathway. The observation of whether treatment with brentuximab vedotin can abrogate this functional T cell impairment, and whether this effect if observed is correlated with clinical response, will provide further data both with regard to potential off target effects of the drug combination, and suggestions for additional rational drug combinations.

1.9.3 SIGNIFICANCE OF INTERLUEKIN-10 INTERLEUKIN-6 IN HL

Interleukin-10 (IL-10) is an immunosuppressive cytokine, which promotes a T-helper-2 (Th2) response, and is secreted by both HRS cells and tumor infiltrating lymphocytes (TILS) in HL. Interleukin-6 (IL-6) is a similar immunosuppressive cytokine. Elevated pre-treatment serum levels of IL-10 and IL-6, have been noted in HL patients compared to normal volunteers in a small study. Both cytokines appeared to decline with successful treatment. High pre-treatment serum IL-6 appeared to be a biomarker of poor response to treatment (32). In a Phase 2 study with brentuximab vedotin as a single agent, IL-10 levels declined in responding patients (Seattle Genetics, private communication). We will evaluate whether IL-10 and IL-6 levels decline as a function of response to therapy with brentuximab vedotin and bendamsutine.

Sequential measurements of PD-1, Tarc, IL-10, and IL-6 may have a role not only in monitoring response to therapy, but also in delineating the immune response to therapy and the monitoring of high risk or minimal residual disease states. This may facilitate both risk stratification and the tailoring of therapy in future novel clinical trials in HL.

2.0 OBJECTIVES

2.1 Primary Objectives: Phase 1

- To determine the maximum tolerated dose (MTD) of brentuximab vedotin and bendamustine
- To determine the dose limiting toxicities (DLT) of brentuximab vedotin and bendamustine

2.2 Secondary Objectives: Phase 1

- To evaluate the overall response rate (ORR) (complete response (CR) + partial response (PR)) for all patients
- To estimate the progression free survival (PFS) and duration of response (DOR) for all patients

2.3 Primary Objective: Phase 2

- To determine the ORR (CR + PR) for the combination of

`

brentuximab vedotin and bendamustine

2.4 Secondary Objectives: Phase 2

- To confirm the safety and tolerability of the combination of brentuximab vedotin and bendamustine
- To evaluate the DOR, PFS and OS for the combination of brentuximab vedotin and bendamustine

2.5 Correlative Studies Phase 1 and Phase 2

- To evaluate serum Tarc levels in patients as a function of treatment with brentuximab vedotin and bendamustine
- To evaluate the level of peripheral blood lymphocyte expression of programmed death-1 (PD-1) as a function of treatment with brentuximab vedotin and bendamustine
- To evaluate the decline in serum levels of IL-10 and IL-6 as a function of treatment with brentuximab vedotin and bendamustine

2.6 Endpoints

2.6.1 Phase 1 Endpoints

- Type, incidence, severity, seriousness, and relatedness of laboratory abnormalities using standard criteria (CTCAE v4.0, Common Terminology Criteria for Adverse Events [http://evs.nci.nih.gov/ftp1/CTCAE/About.html])
- Type, incidence, severity, seriousness, and relatedness of adverse events (CTCAEv4.0)

2.6.2 Phase 2 Endpoints

- ORR (CR+PR)
- Safety, tolerability, OS, PFS, and duration of response

2.6.3 Other Endpoints

- Serum Tarc level pre-treatment, intra-treatment, after completion of treatment, and at relapse (if relapse documented)
- Peripheral blood lymphocyte expression of PD-1 pre-treatment, intra-treatment, after completion of treatment, and at relapse (if relapse documented)
- Serum IL-10 and IL-6 levels pre-treatment, intra-treatment, after completion of treatment, and at relapse (if relapse documented)

3.0 INVESTIGATIONAL PLAN

3.1 Summary of Study Design

This is a phase 1-2 open label, multi-center study to assess the safety and efficacy of brentuximab vedotin in combination with bendamustine in patients with relapsed or refractory HL or anaplastic large cell lymphoma

`

(ALCL). Dose escalation in phase 1 will proceed according to a standard 3 + 3 dose escalation design. There will be no intra-cohort dose escalation. Three patients will be assigned to the starting dose level 1. If no DLT is observed after one cycle of treatment, and if the start of cycle 2 treatment is not delayed greater than 7 days for any toxicity possibly related to drug, trial accrual proceeds to the next dose level and another cohort of 3 patients is enrolled. If 1 patient in a cohort experiences a DLT, then the cohort is expanded to 6 patients. If 2 of 3 or more patients in a cohort experience a DLT then the cohort will be designated the maximum administered dose (MAD) and the dose level below will be expanded. In no more than 2 of 6 patients experience a DLT, this dose level will be designated the MTD. If none of the additional patients experience a DLT (1 out of 6) then dose escalation continues. The MTD is defined as the highest dose level at which <33% of the dose cohort (0 of 3 or 1 of 6) experience a DLT in the first cycle of therapy. Up to a maximum of 10 patients) will be added at the MTD to more fully characterize the safety of the drug combination. As long as <33% of patients experience a DLT, this dose cohort will remain the MTD.

The phase 2 component will employ a 2-stage Simon design (optimum and minimax) that includes a first stage with 19 patients, and a total of 37 patients in the combined stages of the phase 2. With 19 patients in the first stage, the trial will be terminated if there are 12 or fewer responses after 6 cycles of therapy. If the trial proceeds to the second stage (> 12 responses in the first 19 patients) 18 additional patients will be enrolled for a total of 37 patients in the phase 2. The projected enrollment for this entire phase 1-2 study is 59 patients; if all cohorts are expanded at phase 1 this study may treat a maximum of 71 patients.

Brentuximab vedotin will be administered as an outpatient IV infusion on day 1 of each 21-day cycle. Bendamustine will be given as an outpatient IV infusion on days 1 and 2 of a 21 day cycle. Patients may receive prophylactic pegfilgrastim on day 3 of any subsequent cycle after cycle 1, or filgrastim for 5 to 10 days, per investigator's discretion. Patients can receive no more than 6 cycles of this therapy. See Table 1: Drug Administration Schema below.

Table 1: Drug Administration Schema

**Day**	**1**	**2**	**3**	**4-21**
**Brentuximab Vedotin IV**	↓			**Rest**
**Bendamustine IV**	↓	↓		
**Neulasta SQ* (per investigators discretion**			↓	

**after cycle 1)**				

Following the first cycle of therapy disease status will be evaluated using standard criteria. The safety of brentuximab vedotin will be monitored throughout the trial via laboratory tests and adverse event (AE) collection at routine visits. Antitumor activity will be assessed at the end of cycles 2 and 6. Response determination will be based on the Revised Response Criteria for Malignant Lymphoma (33).

Serum and plasma samples will be obtained for the correlative studies as described in section 7.5 End of Treatment (EOT) assessments will be performed within 4-6 weeks after the final dose of study drug. Long-term follow-up contact including survival and disease status will be conducted in a minimum of 3-month intervals from the EOT assessment until either patient death (overall survival) or initiation of additional anti-lymphoma treatment or death from any cause (progression free survival), whichever comes first.

`

An overall schema of events is provided below.

Figure 1: Study Schema

Screening
History, Physical, Performance Status
Blood tests, PET/CT scan, Confirm pathology and CD30 expression

↓

CYCLE 1
<u>*Intervention*</u>
Brentuximab vedotin administered intravenously on day 1 of 21 day cycle
Bendamustine administered intravenously on days 1 and 2 of 21 day cycle
<u>***Evaluations***</u>
Safety evaluation on days: 1, 2, 8, 15
Laboratory evaluation days: 1, 2, 8, 15
Research blood draw days: 1, 8

↓

Future Cycles
<u>*Intervention*</u>
Brentuximab vedotin administered intravenously on day 1 of 21 day cycle
Bendamustine administered intravenously on days 1 and 2 of 21 day cycle
<u>***Evaluations***</u>
Safety evaluation on day 1 and 2 of each cycle
Laboratory evaluation day 1 of each cycle
Research blood , on day 1 of cycles 2 &4 and at EOT (within 14 days of last dose)
-PET/CT or CTscan after cycles 2 & 6 (or last cycle of treatment), and thereafter every 3 to 6 months until disease recurrence or initiation of additional anti-lymphoma treatment

↓

- Disease progression
- Intercurrent illness that prevents further administration of treatment
- Unacceptable adverse event(s), DLTs
- Pregnancy
- Patient decides to withdraw from the study
- General or specific changes in the patient's condition render the patient unacceptable for further treatment in the judgment of the investigator

3.2 Rationale for Selection of Doses and Treatment Schedule

The 1.2mg/kg starting dose of brentuximab vedotin for this study, is the dose of brentuximab vedotin used in the drug-drug interaction study (SGN35-008) in combination with ketaconazole. While not the MTD of brentuximab vedotin as a single agent, the dose of 1.2mg/kg has been associated with meaningful efficacy in the phase 1 study with minimal toxicity.

The starting dose of bendamustine in this study will be 70 mg/m^2/day x 2 days. The FDA approved dose for bendamustine in lymphoma is 120
`

mg/m^2. However, in combination with rituximab, several studies report high response rates ($\geq$ 70%) at a dose of 90mg/m^2, and efficacy of bendamustine in combination with vincristine and prednisone has been reported in low grade lymphoma at doses as low as 60 mg/m^2 (34).

The target dose of brentuximab vedotin in this study is 1.8 mg/kg, which is the brentuximab vedotin dose selected for the pivotal phase 2 trial in HL. The target dose of bendamustine is 100 mg/m2, which is lower than the single agent FDA approved dose for NHL, but consistent with the dose of bendamustine used in the German low grade lymphoma group studies of 90mg/m^2 (23), and the FDA recommended dose for first line treatment of CLL.

Both drugs will be administered on a 21 day schedule. Brentuximab vedotin is given on a 21 day schedule. Bendamustine has been given on a 21 or 28 day schedule in previous protocols, and will be given on a 21 day schedule in this study. The schedules of administration for both drugs will be synchronized.

4.0 STUDY POPULATION

The study population includes patients with relapsed or refractory HL and ALCL.

4.1 Eligibility Criteria

- Histologically confirmed relapsed or refractory HL or ALCL
- Documented CD30+ expression from either original diagnosis or a tumor biopsy in the relapsed setting.
- For patients with HL or ALCL, subjects are eligible after failure of at least one prior multi-agent chemotherapy regimen
- Must have received first line chemotherapy. No upper limit for the number of prior therapies
- Patients with prior autologous or allogeneic stem cell transplant are eligible as long as they meet all other criteria.
- Measurable or evaluable disease, as defined in 2008 Revised Response Criteria for Malignant Lymphoma(33)
- Age $\geq$ 18 years
- ECOG performance status 0,1 or 2
- Patient's must have adequate organ and marrow function as defined below
  - Absolute neutrophil count $\geq$ 1,000 (1.0 **x** 10^9/L)
  - Platelets $\geq$ 50,000 (50 x 10^9/L)
  - Total Bilirubin $\leq$ 1.5 x institutional limits unless documented Gilbert's syndrome (then <2.5 x institutional upper limit)
  - AST (SGOT)/ALT (SGPT) $\leq$ 2.0 x institutional upper limit of normal (unless known hepatic involvement then < 3.5 x institutional upper limit)
  - Creatinine within normal institutional limits OR creatinine clearance $\geq$ 50mL/min for patients with creatinine levels above institutional normal
- If female of childbearing age, negative serum pregnancy test within 7 days prior to the first dose of brentuximab vedotin in this study
- Must be willing to use contraception during the study, and
`

for 30 days following the last dose of study drug.

- Able to understand and to sign a written consent document

4.2 Exclusion Criterion

- Prior treatment with brentuximab vedotin and bendamustine **in combination**. May have received prior therapy with brentuximab vedotin or bendamustine separately.
- Received either brentuximab vedotin or bendamustine within 3 months of receiving their first dose of protocol based therapy.
- If brentuximab vedotin or bendamustine was previously received, had disease progression during the first 3 cycles of either brentuximab vedotin or bendamustine.
- Systemic steroids that have not been stabilized to the equivalent of $\leq$ 10 mg/day of prednisone 7 days prior to the initiation of the trial
- ANY concurrent investigational agents
- Exposure to chemotherapy, radiotherapy, biologics or investigational agents within 3 weeks prior enrollment in the study
- Known cerebral or meningeal disease
- Active concurrent malignancy (except non-melanoma skin cancer or carcinoma in situ of the cervix). If there is a history of prior malignancy the patients must be disease free and off treatment for $\geq$ 3 years.
- Uncontrolled intercurrent illness including but not limited to: ongoing or active infection, systemic congestive heart failure Class III or IV by NYHA criteria, unstable angina pectoris, or cardiac arrhythmia, or in patients status post allogeneic transplantation with uncontrolled graft versus host disease (GVHD).
- Pre-existing neuropathy grade III or greater
- Pregnant or nursing
- Known hypersensitivity to brentuximab vedotin, benadmustine, or mannitol
- Known Human Immunodeficiency Virus (HIV) positive, hepatitis A, hepatitis B or hepatitis C; if hepatitis $B_{surface}$ antigen positive or B_{core} antibody positive must have normal liver function tests and be willing and able to take anti-hepatitis medication such as lamivudine or equivalent.

4.3 Inclusion of Woman and Minorities

Both men and woman of all races and all ethnic groups are eligible for this trial

4.4 Removal of Patients from Therapy or Assessment

The reason(s) for withdrawal from study treatment or from the study must be documented in the patient's medical records and case report form (CRF). The investigators will make every reasonable effort to keep each patient on study until all planned treatments and assessments have been performed. Final treatment assessments will be performed before any other therapeutic intervention, if possible.

`

4.5 Criteria for Removal from Study

Patients will be removed from treatment when any of the criteria listed in Section 4.6 apply. The reason for study removal and the date the patient was removed must be documented in the Case Report Form.

Participation in this study is voluntary. Subjects/patients may withdraw at any time or be dropped from the study at the discretion of the investigator should any untoward effects occur or withdrawal is thought in the best interest of the patient. In addition, a subject/patient may be withdrawn by the investigator if he/she violates the study plan or for administrative and/or other safety reasons. The investigator or study coordinator will notify the appropriate parties within 2 business days when a subject has been discontinued/ withdrawn due to an adverse event (Section 8). When a subject discontinues/withdraws prior to study completion, all applicable activities scheduled for the final study visit should be performed at the time of discontinuation. Any adverse events present at the time of discontinuation/withdrawal will be followed until resolution.

4.6 Early Discontinuation of Study Drug

The reason and justification for patient discontinuation of treatment must be documented in the CRF. A patient may discontinue the study under any of the following circumstances:

- Patient decision
- Disease progression
- Clinically significant unresolved adverse event
- Physician decision (i.e. for medical reasons, or patient non-compliance)

4.7 Replacement of Withdrawn Patients

In Phase 1, patients who withdraw from study prior to receiving all planned therapy of cycle 1 for reasons other than a clinically significant adverse event (excluding progression of disease) may be replaced. Patients in Phase 2 who have completed less than 2 cycles of therapy, and who have not had discontinued therapy for reason of disease progression or toxicity, may be replaced. All patients who receive any dose of study drug will be evaluable for toxicity.

5.0 TREATMENT PLAN

This study is being performed under IND application number 115,338.

5.1 Agent Administration and Schedule

Brentuximab vedotin will be administered as an outpatient IV infusion on day 1 of each 21-day cycle. Bendamustine will be given as an outpatient infusion on days 1 and 2 of each 21 day cycle. Growth factor support will not be given in cycle 1, but after cycle one patients may receive prophylactic pegfilgrastim on day 3, or filgrastim for 5 to 10 days, per investigator's discretion.

`

Patients will receive a maximum of 6 cycles of therapy (Table 1). No investigational or commercial agents or therapies other than those described below may be administered with the intent to treat the patient's malignancies. Missed doses of either drug will not be made up. Up to a 3 week delay may be allowed for receiving treatment, excluding cycle 1 in the Phase I or at any time during Phase II.

Table 1: Drug Administration Schema

**Day**	**1**	**2**	**3**	**4-21**
**Brentuximab vedotin IV**	↓			**Rest**
**Bendamustine IV**	↓	↓		
**Neulasta SQ*** **after Cycle 1, per investigators discretion**			↓	

5.2 Brentuximab Vedotin Administration

5.2.1 DESCRIPTION

Brentuximab vedotin is an antibody-drug conjugate consisting of the anti-CD30 antibody cAC10 conjugated to MMAE, an anti-tubulin agent. Brentuximab vedotin is supplied as sterile, preservative-free, white to off-white lyophilized cakes for reconstitution for IV administration and is supplied by Seattle Genetics in single-use glass vials. Each vial contains brentuximab vedotin, trehalose, sodium citrate, and polysorbate 80. See the Pharmacy Manual for further information.

5.2.2 DOSE AND ADMINISTRATION

Brentuximab vedotin is administered as a single outpatient IV infusion on Day 1 of each 21-day treatment cycle. The doses of brentuximab vedotin used in this study are 1.2 mg/kg (dose levels -1-2) and 1.8 mg/kg (dose levels 3-5).

Dosing should be based on baseline weight; however, doses will be adjusted for patients who experience a ≥10% change in weight from baseline. Actual weight will be used for dosing bendamustine based on "Dosing of Treanda in Obese Patients" communication from Cephalon (Appendix 1). For dosing of brentuximab vedotin in patients weighing greater than 100 kg, the dose will be calculated based on 100 kg. The dose will be rounded to the nearest whole number of milligrams. **Study treatment must not be administered as an IV push or bolus.** Study treatment will be administered by outpatient IV infusion given over approximately 30 minutes on Day 1 of each 21-day cycle. In the absence

of infusion toxicities, the infusion rate for all patients must be calculated in order to achieve a 30-minute infusion period. Study treatment will be administered through a dedicated IV line and cannot be mixed with other medications. The administration of brentuximab vedotin in relation to meals is not specified. For required premedication, prophylaxis, and the management of infusion reactions see section 5.4 below.

5.2.3 Storage and Handling

Vials containing brentuximab vedotin must be refrigerated at 2–8°C in an appropriate locked room accessible only to the pharmacist, the investigator, or a duly designated person. Reconstituted brentuximab vedotin should be used immediately; if not used immediately, in-use storage times should not be longer than 24 hours at 2–8°C. The effect of light on brentuximab vedotin has not been assessed therefore it is recommended that brentuximab vedotin vials and solutions be stored in the dark until the time of use. Reconstituted vials must not be shaken. Drug accountability instructions are provided in the Pharmacy Manual.

5.2.4 Packaging and Labeling

Brentuximab vedotin is the United States adopted name (USAN) assigned to SGN-35. Vialed drug product may be labeled as SGN-35, brentuximab vedotin, or as Adcetris. The 3 names can be used interchangeably. Refer to the Pharmacy Manual for additional information regarding packaging and labeling.

5.2.6 Preparation

Brentuximab vedotin vials are provided via single-use containers. Any partially used vials or diluted dosing solutions are to be discarded using appropriate institutional drug disposal procedures. Brentuximab vedotin must be reconstituted with the appropriate amount of Sterile Water for Injection, USP (see Pharmacy Manual for details). GENTLY swirl the vial until the contents are completely dissolved. **The vial must not be shaken or vigorously swirled**; excess agitation may cause aggregate formation. Visually inspect the reconstituted drug product for any particulate matter and discoloration. The appropriate amount of reconstituted brentuximab vedotin will be withdrawn from the vial(s) and diluted in a 100mL or 150mL infusion bag containing 0.9% Sodium Chloride Injection, USP. There are no known incompatibilities between brentuximab vedotin and polyvinylchloride bags. The bag should be gently inverted to mix the solution. **The bag must not be shaken** as excess agitation may cause aggregate formation. Prior to administration, the reconstituted and diluted drug product should be inspected visually for any particulate matter and discoloration.

5.3 Bendamustine Administration

5.3.1 Description

Bendamustine is a unique bifunctional agent with an alkylating mechlorethamine group conjoined to

`

a purine like benzamidazole ring. Bendamustine is supplied as lyophilized powder from Celphalon, Inc. in single use vials containing 100mg of lyophilized powder.

5.3.2 Dose and Administration

Bendamustine will be administered by outpatient IV infusion given over approximately 60 minutes on days 1 and 2 of a 21 day cycle. Study treatment will be administered through a dedicated IV line.

Dosing of bendamustine should be based on baseline weight. Doses will be adjusted for patients who experience a ≥10% change in weight from baseline. Actual weight will be used for all patients. The dose will be rounded to the nearest whole number of milligrams.

5.3.3 Storage and Handling

Bendamustine should be stored and handled as specified in the package insert.

5.4 Required Premedications and Prophylaxis

Routine premedication should not be administered prior to the first dose of brentuximab vedotin. However, patients who experience a Grade 1 or Grade 2 infusion-related reaction with brentuximab vedotin may receive subsequent brentuximab vedotin infusions with premedication consisting of acetaminophen (650 mg orally) and diphenhydramine (25–50 mg orally or 10–25 mg IV), administered prior to each 30-minute infusion, or a suitable alternative according to institutional standards or investigator discretion. Patients who experience a Grade 3 or Grade 4 brentuximab vedotin infusion-related reaction may potentially receive additional treatment at the discretion of the investigator.

Routine anti-emetic pre-medication with bendamustine infusion should be administered according to institutional standards or investigator discretion. Growth factor support will not be allowed in cycle 1, but after cycle one patients may receive prophylactic pegfilgrastim on day 3, or filgrastim for 5 to 10 days, per investigator's discretion. Patients will receive a maximum of 6 cycles of this therapy. For patients who are post allogeneic SCT standard international guidelines for infection prophylaxis for herpes simplex virus (HSV), varicella-zoster virus (VZV), and *Pneumocystis jiroveci* (PCP) should be followed. At the investigator's discretion similar anti-infection prophylaxis may be used for post autologous SCT patients. See Table 2: Regimen Premedication Description below.

Table 2: Regimen Premedication Description.

**REGIMEN DESCRIPTION**

`

Agent	Premedications; Prophylaxis	Dose	Route	Schedule	Cycle Length
Brentuximab Vedotin	cotrimoxazole 1 DS tablet three times weekly; Acyclovir (optional); Acetaminophen and Diphenhydramine (optional)	1.2-1.8mg/kg**	IV infusion over 30 minutes	Day 1	21- days
Bendamustine	Anti-emetics, Neulasta on Day 3*	60-100 mg/m2**	IV infusion	Days 1 and 2	
*After cycle 1 per investigator's discretion<br>** Doses as appropriate for assigned dose level.					

5.4.2 Management of Infusion Reactions

Infusion-related reactions may occur during the infusion of brentuximab vedotin. The infusion is to be administered at a site properly equipped and staffed to manage anaphylaxis should it occur. On cycle 1, day 1 only, the patient should be observed for at least 60 minutes following the end of the first infusion of the treatment (brentuximab vedotin and bendamustine). During this observation period, the IV line should remain open for at least 1 hour to allow administration of IV drugs if necessary. All supportive measures consistent with optimal patient care will be given throughout the study according to institutional standards. This includes adjusting the infusion time if necessary. Medications for infusion-related reactions, such as epinephrine, antihistamines, and corticosteroids, should be available for immediate use.

Patients who experience a Grade 1 or Grade 2 infusion-related reaction may receive subsequent brentuximab vedotin infusions with premedication consisting of acetaminophen (650 mg orally) and diphenhydramine (25–50 mg orally or 10–25 mg IV), administered prior to each 30-minute infusion, or a suitable alternative according to institutional standards or investigator discretion.

5.5 Phase 1 Dose Escalation and Reduction Rules

Dose escalation in phase 1 will proceed according to a standard 3 + 3 dose escalation design, as shown in Table 3. Three patients will be assigned to the starting dose level 1. If no DLT is observed after 3 patients have received 1 cycle of therapy, and if the administration of cycle 2 is not delayed for greater than 7 days for toxicities possibly related to drug, the trial will proceed to the next dose level and another cohort of 3 patients will be enrolled. If 1 patient in any cohort experiences a DLT then the cohort is expanded to 6 patients. If > 2 patients out of 6 experience a DLT, then this dose level is declared the maximum acceptable dose (MAD), and there will be no further dose escalation. The MTD is defined as the highest dose level at which <33% of the dose cohort (1 of 3 or <2 of 6) experience a DLT in the first cycle. Up to an additional 4 patients can be added at the MTD level to more fully characterize the safety of the combination (total of 10 patients

`

will be treated at the MTD). As long as ≤3 patients in this expanded cohort experience a DLT, this dose level will be declared the recommended phase 2 dose level (RP2D). If more than 3 patients experience a DLT, we will and adopt this dose level as the MAD and drop to the dose level immediately below.

Table 3: Dose Escalation Schema

Dose Level	Brentuximab Vedotin(mg/kg)	Bendamustine (mg/m2)
Level -1	1.2	60
Level 1	1.2	70
Level 2	1.2	80
Level 3	1.8	80
Level 4	1.8	90
Level 5	1.8	100

The dose-escalation rules and determination of the maximum tolerated dose (MTD) are outlined below. Patients will receive sequentially escalating dose of brentuximab vedotin and bendamustine until a MTD has been defined. The MTD of brentuximab vedotin in combination with bendamustine will be defined at the highest dose at which ≤1 of 3 or ≤2 of 6 patients experience a DLT. The maximum tolerated dose of brentuximab vedotin in combination with bendamustine will be determined based on dose-limiting toxicities that occur during the first cycle of study drug administration.

Table 4: Dose Escalation Decision Rules

The dose level below the MAD should be expanded to 6 patients. If 2 or fewer patients experience a DLT, then this dose level will be considered the MTD.

Number of Patients with DLT at a Given Dose Level	Escalation Decision Rule
0 out of 3	Enter 3 patients at the next dose level.
1 out of 3	Enter at least 3 more patients at this dose level.<br>• If 1 of these 6 patients experience DLT, proceed to the next dose level.<br>• If 2 or more of this group suffer DLT, then dose escalation is stopped, and this dose is declared the maximally administered dose (MAD). If no more than 2 of 6 patients experience a DLT at the next lowest dose level, that will be designated the MTD.

`

≥2	Dose escalation will be stopped. This dose level will be declared the maximally administered dose (highest dose administered). Up to 7 additional patients will be entered at the next lowest dose level if only 3 patients were treated previously at that dose.
≤2 out of 6 at highest dose level below the maximally administered dose	This is the MTD and the recommended phase 2 dose (RP2D).A maximum of 10 patients total in the cohort will be entered at the MTD to more fully characterize the safety of the drug combination.

5.6 Dose De-escalation Decision Rules

If DLT is reached in ≥2 of 6 patients in the first cycle of dose level 1, then the doses of brentuximab vedotin and bendamustine will be reduced to dose level -1, and there will be no further dose escalation. If ≤2 patients have a DLT at dose level -1, this dose level will be defined as the MTD, and become the Phase 2 dose.

Toxicities in cycles 2 and following may be accommodated by dose de-escalation of either drug as detailed in **Table 3**, but toxicities in cycles 2 and following will not affect dose escalation for future patients, as is standard in Phase I trial design.

5.7 Dose-Limiting Toxicity

To constitute a DLT, toxicities should be attributable to the study drugs which occur in cycle 1 only. A delay of 7 or more days in starting cycle 2 will also be considered a DLT. Toxicity will be graded using the Common Toxicity Criteria for Adverse Events (CTCAE) version 4.0. DLT is defined as any toxicity not specifically excluded below that occurs within cycle 1 and is considered at least “possibly related to study drug administration.” For patients who discontinue study drug administration after receiving all planned therapy during Cycle 1, DLT is defined as any toxicity not specifically excluded below that occurs within 26 days of the last dose of drug on study (what would have been 1 complete cycle + 7 days delay in the start of cycle 2.

Patients who only receive Day 1 of cycle 1 and discontinue for reasons other than DLT may be replaced. They will be followed for toxicity, but they will not count towards the DLT determinations nor dose escalation schema.

DLT is defined by any of the following events during the first cycle of treatment:

- Grade 4 neutropenia that does not resolve to ≤ Grade 2 within 7 days or Grade 4 thrombocytopenia lasting more than 7 days
- Febrile neutropenia ≥ Grade 3 as defined by CTCAE version 4.0
- Inability to re-treat patients within a 1 week period of their scheduled treatment due to a treatment related event
- Any other non-hematologic clinical toxicity defined as any Grade 3 or greater toxicity with the specific exception of:

`

- Grade 3/4 nausea or Grade 3/4 vomiting that in the opinion of the investigator occurs in the setting of inadequate premedication or compliance with supportive care measures and lasts for less than 48 hours.
- Grade 3 diarrhea that in the opinion of the Investigator occurs in the setting of inadequate premedication or compliance with supportive care measures and lasts for less than 48 hours.
- Grade 3 dehydration that, in the opinion of the investigator, occurs in the setting of inadequate prophylactic measures or compliance with supportive care measures and lasts for less than 48 hours.

- Alopecia, any grade.
- Grade 3 Constipation that in the opinion of the Investigator occurs in the setting of inadequate prophylactic measures or compliance with supportive care measures and lasts for less than 5 days.
- Grade 3 Fatigue that lasts for ≤ 5 days
- Grade 3 or 4 lymphopenia

5.8 Dosing Delays and Dose Modifications

5.8.1 Dose Delays

- Study drugs can be administered within 48 hours of their scheduled time, excluding cycle 2 in phase 1.
- Study drugs will be held for DLTs until adverse event returns to ≤ Grade 2
- If interruption lasts for more than 21 days, study treatment will be discontinued.

5.8.2 Dose Modifications

No dose modifications of brentuximab vedotin or bendamustine are allowed in this study without prior consultation with the Principle Investigator. After cycle 1, subsequent cycles of brentuximab vedotin and bendamustine may be delayed for up to 3 weeks if additional time is required for the patient to recover from toxicity. Delays of greater than 3 weeks are prohibited and will lead to discontinuation of study participation. For toxicity-related modifications beyond cycle 1, intra-patient dose reduction will be allowed depending on the type and severity of toxicity. **Table 5** describes dose modifications for toxicity associated with brentuximab vedotin and/or bendamustine beyond cycle 1.

Table 5: **Dose Modifications for Toxicity with Brentuximab Vedotin or Bendamustine Beyond Cycle 1**

Toxicity	Grade 1	Grade 2	Grade 3	Grade 4

`

**Non-Hematologic**	Continue at same dose level	Continue at same dose level, except in the event of Grade 2 neuropathy. For Grade 2 neuropathy, withhold dose of brentuximab vedotin until toxicity is ≤ Grade 1 or has returned to baseline, then reduce the dose to 1.2 mg/kg and resume treatment.	For both brentuximab vedotin and bendamustine withhold dose until toxicity is ≤ Grade 1 or has returned to baseline, then resume treatment at the same dose level. For Grade 3 or higher neuropathy, discontinue treatment.	For both drugs withhold dose until toxicity is ≤ Grade 1 or has returned to baseline, then reduce brentuximab vedotin dose to 1.2 mg/kg and bendamustine dose by 25% and resume treatment.
**Hematologic for neutrophils (ANC) and platelets only**	Continue at same dose level	Continue at same dose level	For both drugs withhold dose until toxicity is ≤ Grade 2, or has returned to baseline, then resume treatment at the same dose level.	For both drugs withhold dose until toxicity is ≤ Grade 2, then resume treatment at the same dose level. For the second occurrence of Grade 4 toxicity withhold dose until toxicity is ≤ Grade 2, then reduce the dose of brentuximab vedotin to 1.2 mg/kg and bendamustine by 25% and resume treatment

5.8.3 Resuming Administration of Study Drug

For toxicities that can be treated or prevented, such as nausea, vomiting, diarrhea and neutropenia, treatment may be resumed at the previous dose once supportive measures have been instituted and toxicity recovers to Grade 2 or less.

5.9 Concomitant Therapy

All concomitant medications and blood products administered for each patient beginning 4 weeks prior to first dose of study drug and continuing through the EOT visit must be recorded in the source document and on the CRF. Supportive measures consistent with optimal patient care should be provided throughout the study according to institutional standards.

5.9.1 Required Concomitant Therapy

No required concomitant therapy other than the routine prophylaxis specified above in section 5.4 is required on this study.

5.9.2 Prohibited Concomitant Therapy

No other anti-cancer treatment will be permitted during the treatment period, including chemotherapy, investigational therapies, radiation, biological response modifiers, hormone therapy, or immunotherapy. Systemic steroids in the excess of 10mg/day, or which have not been stabilized > 7 days before the start of therapy are prohibited. Immunosuppressive therapy as treatment for or prophylaxis against GvHD is prohibited.

5.10 Treatment Compliance

Subject compliance with treatment is anticipated to be well controlled as
`

study treatment will be administered at the study site by the treating physician investigator. Treatment administration data will be captured and reviewed to ensure site compliance with the treatment regimen.

5.11 Treatment Duration

The maximum number of treatments with brentuximab vedotin and bendamustine is 6 cycles of therapy. Patients may discontinue therapy prior to completing 6 cycles if the following criterion applies:

- Patient decision
- Disease progression
- Clinically significant unresolved adverse event
- Physician discretion (i.e. for medical reasons, or patient non-compliance).

6.0 STUDY ACTIVITIES

6.1 Study Procedures

Each patient must sign and date an informed consent form before undergoing any study specific procedure unless a procedure is being performed as part of the patient's standard of care. Enrollment in the study requires that all inclusion and exclusion criteria have been met. Enrollment in the study is defined when the written consent is obtained, confirmation that the patient meets all inclusion and exclusion criteria has been obtained, and the completed enrollment form has been signed.

Concomitant medications, concomitant procedures, and adverse events will be collected from the time of informed consent through the EOT visit or 4 to 6 weeks after the last dose, (whichever is later). A detailed schedule of events, including acceptable windows for performing assessments in outlined in the Schedule of Events in Section 6.4 below.

6.2 Registration Procedures

6.2.1 General Guidelines

Eligible patients will be entered on study by the study staff at Columbia University Medical Center.

Following registration, patients should begin protocol treatment within 7 days. Issues that would cause treatment delays should be discussed with the Principal Investigator. If a patient does not receive protocol therapy following registration, the patient's registration on the study may be canceled.

6.2.2 Screening

All potential study patients will be screened and eligibility will be determined prior to enrollment.

`

Unless otherwise specified, the following procedures and evaluations will be performed as noted in the study calendar prior to the start of study drugs in Cycle 1.

1. Obtain written informed consent and privacy authorization prior to initiating any protocol-required procedure that is not considered standard of care (See Section xx)
2. Review eligibility criteria
3. Review medical chart for past medical/surgical history
4. Record medications and prior treatment regiments
5. Record response to prior treatment regiments
6. Document histopathology of HL or ALCL including CD30+ expression from either:
    - Original diagnosis
    - Tumor biopsy in the relapsed setting
7. Documentation of known measureable disease by the following parameters:
    - Whole body PET/CT or CT of neck, chest, abdomen, and pelvis
8. Obtain a 12-lead electrocardiogram (ECG) and calculate the QTcB interval.
9. Perform a comprehensive physical examination
10. Assess and record ECOG Performance Status
11. Routine laboratory studies including:
    - Complete blood count with differential counts
    - Serum chemistry including liver function tests
    - Serum β-human chorionic gonadotropin [β-hCG] pregnancy test for women who are not postmenopausal or surgically sterile (within 7 days prior to cycle 1, dose 1)
    - Creatinine clearance using the glomerular filtration rate (GFR) according to the Cockroft-Gault Equation
    - Serum hemoglobin A1C
12. Patients post-allogeneic bone marrow transplant only
    - CMV pcr within 8 days of enrollment in the clinical trial

6.3 Registration Process

To register a patient, the following documents should be completed by the research nurse or data manager and sent to the study coordinator at CUMC:

- Eligibility Screening Worksheet
- Copy of required laboratory and imaging tests
- Signed patient consent forms
- HIPAA authorization form

The Study Coordinator will verify eligibility. To complete the registration process, the Coordinator will:

- Assign a patient study number
- Register the patient on the study
- Confirm registration with the principal investigator

`

6.4 Schedule of Events

A detailed schedule of patient visits, and study assessments is outlined in the study calendar below.

Figure 2: Study Calendar

Cycle		Cycle 1				Cycles 2 and 3	Cycle 4	Cycles 5 and 6	End of Study	Survival Follow-up
	Screening[b]	Wk 1 Day 1	Wk 1 Day 2	Wk 2 Day 8	Wk 3 Day 15	Wk 1 Day 1	Wk 1 Day 1	Wk1 Day 1	4 to 6 weeks after last dose	
Eligibility & Safety Monitoring										
Informed Consent	X									
Demographics	X									
Medical History	X									X
Concurrent Medications	X	X		X	X	X	X	X	X	X
Physical exam	X	X		X	X	X	X	X	X	X
Vital Signs	X	X	X	X	X	X	X	X	X	X
Weight	X	X				X	X	X	X	
Bone Marrow Biopsy	X[j]									
Lymph Node Biopsy	X[j]									
Performance Status	X	X	X	X	X	X	X	X	X	X
CBC w/ differential	X	X	X	X	X	X	X	X	X	X
Chemistries[a]	X	X		X	X	X	X	X	X	X
Hemoglobin A1C	X									
CMV PCR[c]	X					X	X	X	X	
EKG	X									
Toxicity Assessment		X		X	X	X	X	X	X	X
Serumβ-hCG	X									
Cycle		Cycle 1				Cycles 2 and 3	Cycle 4	Cycles 5 and 6	End of Study	Survival Follow-up
	Screening[b]	Wk 1 Day 1	Wk 1 Day 2	Wk 2 Day 8	Wk 3 Day 15	Wk 1 Day 1	Wk 1 Day 1		Screening[b]	Wk 1 Day 1
Urinalysis	X					X	X	X	X	
Efficacy Measurements										
CT or PET/CT	X					X[d]			X	X[e]
Correlative Blood Draw[f]		X		X		X[g]	X		X	X[h] and X[i]
All evaluations within +/- 2 days in cycle 1 and +/- 4 days thereafter										

a: creatinine, bilirubin, AST, ALT, alkaline phosphatase, sodium, potassium, chloride, HCO_3, LDH, calcium, ESR
b: screening within 8 days of study entry except Informed consent within 28 days and CT or PET/CT within 30 days.
c: only in patients s/p allogeneic bmt
d: repeat CT or PET/CT prior to starting cycle 3 e: for patients with CR, PR, or SD, CT or PET/CT every 3 to 6 months until relapse and/or subsequent therapy per treating physician. Survival follow-up may be conducted by telephone if patient does not live locally.
f: blood draws pre-treatment serum and plasma for correlative studies
g: cycle 2 only
h: at time of POD
i: with consent at other intervals concomitant with regular follow-up
j: only if clinically necessary to confirm recurrent disease
k: Within 7 days of Cycle 1 Day 1.

`

6. 5 Required Blood Parameters and Other Investigations Prior to Each Treatment

Before the start of each treatment, patients should be reassessed and the following criteria must be fulfilled:

- Absolute neutrophil count ≥ 1,000 (1.0 x 10^9/L)
- Platelets ≥ 50,000 (50 x 10^9/L)
- Serum creatinine concentration ≤ 2.0 × ULN or ≤ baseline
    - AST (SGOT) and ALT (SGPT) ≤2.0 × ULN (unless known hepatic involvement then < 3.5 x institutional upper limit)
-
    - Total Bilirubin concentration ≤2.0 × ULN (unless documented Gilbert's syndrome then <2.5 x institutional upper limit)
- Recovery of any drug-related non-hematological toxicity to Grade 1 or less, unless otherwise indicated

6.6 End of Study Visit & Follow up visits

Patients will have a follow up appointment 4 to 6 weeks after the last dose of therapy for lymphoma and toxicity assessment. Patients removed from study for unacceptable adverse events will be followed until resolution or stabilization of the adverse event**.** Medications, adverse events, and disease status will be recorded for each subject at that time.

For patients who have stable disease or better after 6 cycles of therapy will be further followed every three months after the 4-week safety evaluationLymphoma assessment will be performed by medical history, physical, CBC with differential and chemistries every 3 months until progression of disease occurs. PET/CT or CT will be performed every 3 to 6 months until disease progression occurs, or the patient initiates a new therapy, per the discretion of the treating physician. For patients who live at a distance from a study center, this follow-up may be conducted by telephone.

7.0 STUDY ASSESMENTS

7.1 Screening/Baseline Assessments

See sections 6.2.2., 6.4, 6.5, 7.2 and 7.3

7.2 Safety Assessments

The assessment of safety during the course of this study will consist of the surveillance and recording of adverse events (AEs) including serious adverse events (SAEs), recording of concomitant medications and measurement of protocol-specified physical examination findings and laboratory tests, according to the time points outlined. Safety will be monitored over the course of the study.

7.3 Lymphoma Assessments

Clinical assessments are to be performed at the timepoints outlined

`

in the schedule of events. Every effort must be made to perform clinical assessments according to the schedule of events to prevent the introduction of bias based on treatment delays.

An adequate focused clinical assessment consists of:

- Interval patient medical history including a thorough review of signs and symptoms, including B symptoms
- Concomitant medications
- Physical Examination (including evaluation of skin, HEENT (head, eyes, ears, nose and throat) lymph nodes, heart, lungs, abdomen, back, extremities, and neurology
- Laboratory values including chemistry panel, liver panel, hematology panel, and Erythrocyte Sedimentation Rate (ESR)

Radiographic assessment, either a full IV contrast CT of neck/chest/abdomen/pelvis alone or in combination with a PET scan (PET/CT), will be performed at protocol specific time-points specified above in section 6.4, or at any time-point if disease progression is suspected.

Assessment of lymphoma response (CR, PR or SD) and disease progression will be evaluated as outlined in the schedule of events, according to the Revised Response Criteria for Malignant Lymphoma (33). A full IV contrast CT scan combined with a PET scan is preferred for baseline evaluation, interim evaluation, and at the completion of study, but the PET component is not mandatory. Overall response rate, CR, PR, SD, PFS and DOR will be calculated as detailed in section 10, the statistical analysis section.

7.4 Other Study Assessments

Other study assessments include the following:

- Tissue from the original and/or most recent post-diagnostic biopsy of relapsed/refractory disease must be submitted to the treating physician for confirmation of relapsed Hodgkin lymphoma or ALCL via slides or tumor block, if the tissue has not already been reviewed by the study physician’s hospital.
- Comprehensive patient medical history, including a thorough review of:
    - The patient’s current and previous conditions.
    - Disease status after each previous systemic therapy received prior to entering this study.
    - Results of previously obtained, post-salvage, pre-transplant PET scan (if done).
    - Concomitant medications and prior therapies, including radiation.
    - Assessment of peripheral neuropathy
    - Electrocardiogram.
    - ECOG performance status

`

7.5 Assessment of Correlative Studies

Validated assays will be used to measure the correlative endpoints. Assays used will include enzyme-linked immunosorbent assays (ELISA) and flow cytometry, as well as other assays if further characterization is required. Assessment of additional biomarkers may be performed at the investigator’s discretion. Immunhistochemistry will be used in the investigation of primary tumor tissue. With patient consent blood samples may also be drawn in follow-up after study completion.

7.5.1 Schedule of Correlative Study Collection

Serum and plasma samples for correlative studies will be collected as specified in the schedule of events and are outlined below. All laboratory assessments are collected before treatment unless otherwise specified.

- Pre-treatment: cycle 1, day 1
- Cycle 1 day 8
- Cycle 2, day 1 pre-dose
- Cycle 4, day 1, pre-dose
- End of study visit
- At time of POD on treatment, or if patient has completed treatment with patient consent
- In follow-up after treatment completion with patient consent (optional) at three month intervals or in conjunction with routine follow-up.

7.5.2 Tarc Evaluation

The assessment of serum Tarc levels will be measured by enzyme-linked immunosorbent assay (ELISA) or by Luminex assay.

7.5.3 PD-1 Evaluation

The level of PD-1 expression on peripheral blood lymphocytes will be evaluated by flow cytometry.

7.5.4 IL-6 and IL-10 Evaluation

The level of serum expression of IL-6 and IL-10 will be measured by ELISA or by Luminex assay

The methods to analyze the immunologic response used in this study are well established. However, as immune regulation is a very active field of research, the opportunity exists to refine and extend the immune analysis. Therefore, the majority of blood drawn for the analysis of the immune response will be used for the assays listed in this section. However other aspects of the T cell response may be explored with these samples as well. These approaches will be exploratory by definition and will not constitute primary endpoints of the study. No patient blood samples collected in this study will be banked after the conclusion of the study analysis, or utilized in future research.

7.6 Handling of Specimens

All correlative samples will be processed by Dr. Owen A.

`

O'Connor's laboratory at CUMC or in the CUMC pathology core facilities or the HICCC core facilities.

8.0 Adverse Events

8.1. Definitions

8.1.1 Adverse Event (AE)

According to the International Conference on Harmonization (ICH) guidelines (Federal Register. 1997;62(90):25691-25709) and 21 CFR 312.32, IND Safety Reports, and ICH E2A, Definitions and Standards for Expedited Reporting, an adverse event is defined as follows:

"An adverse event is any untoward medical occurrence in a subject or clinical investigational subject administered a pharmaceutical product and which does not necessarily have a causal relationship with this treatment. An adverse event can therefore be any untoward medical occurrence regardless of relationship to the medicinal (investigational) product."

Adverse events will be recorded from the time of informed consent. The following information should be considered when determining whether or not to classify a test result, medical condition, or other incident as an adverse event:

- Abnormal laboratory values should not generally be recorded as an adverse event unless an intervention is required, the laboratory abnormality results in a serious adverse event, the laboratory abnormality results in study termination or interruption/discontinuation of study treatment or is associated with clinical signs or symptoms**.** When recording an adverse event resulting from a laboratory abnormality, the resulting medical condition rather than the abnormality itself should be recorded (e.g., record "anemia" rather than "low hemoglobin."

- Medical conditions present at screening (i.e., ongoing at time informed consent is obtained) should be recorded on the Adverse Event/Pre-existing Condition case report form (CRF) so that worsening or improvement of the conditions during the study may be tracked.

- Changes in pre-existing medical conditions, including changes in severity, frequency, or character, during the protocol-defined reporting period should be recorded on the AE CRF.

- Complications that occur in association with a protocol-mandated intervention (e.g., invasive procedures such as biopsies) should be recorded as AEs.

8.1.2 Serious Adverse Event

`

An adverse event should be classified as a serious adverse event (SAE) or adverse drug reaction (AE) if it meets one of the following criteria:

Fatal: Adverse event resulted in death

Life threatening: The adverse events placed the patient at immediate risk of death. This classification did not apply to an adverse event that hypothetically might cause death if it were more severe.

Hospitalization: The AE required or prolonged an existing inpatient hospitalization. Hospitalizations for elective medical or surgical procedures or treatments planned before the signing of informed consent in the study or routine check-ups are not serious adverse events by this criterion. Admission to a palliative unit or hospice care facility is not considered to be a hospitalization. Hospitalizations or prolonged hospitalizations for scheduled therapy of the underlying cancer or study target disease need not be captured as SAEs**.**

Disabling/ incapacitating: Resulted in a substantial and permanent disruption of the patient's ability to carry out activities of daily living.

Congenital Abnormality: Causing a congenital abnormality or birth defect

Medically significant: The adverse event did not meet any of the above criteria, but could have jeopardized the patient and might have required medical or surgical intervention to prevent one of the outcomes listed above.

Infectious Transmission: Any suspected transmission via a medicinal product of an infectious agent.

Adverse Event Severity: AE severity will be graded using the NCI CTCAE, Version 4. These criteria are provided in the study manual, or may be accessed http://ctep.cancer.gov/reporting/ctc.html. AE severity and seriousness are assessed independently. 'Severity' characterizes the intensity of an AE. 'Serious' is a regulatory definition and serves as a guide to the Sponsor for defining regulatory reporting obligations.

Relationship of the Adverse Event to Study Treatment: The Sponsor-Investigator shall use his/her judgment to determine the relationship between the Serious Adverse Event and the Study Drug. The relationship of each adverse event to study treatment brentuximab vedotin and bendamustine should be evaluated by the Investigator using the following criteria:

Related: There is a plausible temporal relationship between the onset of the AE and administration of the study treatment; no alternate
`

etiology for the AE exists; the AE follows a known pattern of response to the study treatment; or the AE reappears upon re-challenge

Unrelated: Another cause of the adverse event is more plausible; a temporal sequence cannot be established with the onset of the adverse event and administration of the study treatment; or, a causal relationship is considered biologically implausible

8.2 Procedures for Eliciting and Recording Adverse Events

Investigators and study personnel will report all AEs and SAEs whether elicited during patient questioning, discovered during physical examination, laboratory testing and/or other means by recording them on the Adverse Event/Pre-existing Condition CRF and/ or SAE form, as appropriate.

8.2.1 Eliciting Adverse Events

An open-ended or non-directed method of questioning should be used at each study visit to elicit the reporting of AEs.

8.2.2 Recording Adverse Events

Regardless of relationship to study treatment, all serious and non-serious adverse events that occur during the protocol-defined AE and/or SAE reporting period are to be recorded on an Adverse Event /Pre-existing Condition CRF during the study.

The following information should be collected for adverse events:

- Description of the adverse event including onset and resolution dates
- Severity of Adverse Event
- Relationship to study treatment or other causality
- Outcome of each event
- Whether event met SAE criteria

The following outcomes are to be recorded in the description of the outcome of AEs:

- Resolved: The patient has fully recovered from the event with no residual effects observable
- Stabilized: Effects of the event are constant. The likelihood of these effects changing (improving or worsening) is low
- Ongoing: Effects of the event are still present and changing. The event is not considered stabilized or resolved.

8.2.3 Diagnosis vs. Signs or Symptoms

`

In general, the use of a unifying diagnosis is preferred to the listing out of individual symptoms. Grouping of symptoms into a diagnosis should only be done if each component sign and/or symptom is a medically confirmed component of a diagnosis as evidenced by standard medical textbooks. If any aspect of a sign or symptom does not fit into a classic pattern of the diagnosis, report the individual symptom as a separate adverse event.

Important exceptions for this study are adverse reactions associated with the infusion of study treatment. For infusion-related reactions, do not use the NCI CTCAE terms of ‘cytokine release syndrome’, ‘acute infusion reaction’ or ‘allergic or hypersensitivity reaction’. Instead, record each sign or symptom as an individual adverse event. If multiple signs or symptoms occur with a given infusion-related event, each sign or symptom should be recorded separately with its level of severity.

8.2.4 Recording Serious Adverse Events

For SAEs, record the primary event on both an Adverse Event /Pre-existing Condition CRF and an SAE form; events occurring secondary to that primary event should be described on the SAE form in the narrative description of the case. For SAEs that occur after informed consent but before any protocol-required drug is dosed and that are considered unrelated to any study procedure by the investigator, record on the Adverse Event /Pre-existing Condition CRF only; an SAE form is not required for such events.

The following should be considered when recording SAEs:

- Results in death. The event that resulted in the death should be recorded and reported on both an SAE form and CRF.
- Is life threatening. This definition implies that the patient, in the view of the investigator is at immediate risk of death from the event as it occurred. It does not include an event that had it occurred in a more severe form, might have caused death.
- Requires inpatient hospitalization, or prolongs existing hospitalization
- Results in persistent or significant disability/incapacity
- Results in a congenital anomaly birth defect in a child of patient who participated in the study and received study drug.
- Other important medical events at the discretion of the investigator which may not be immediately life threatening or result in death or require hospitalization but may jeopardize the subject or require intervention to prevent one of the outcomes listed above.

8.2.5 Pregnancy

All pregnancies that occur within 30 days of last dose of study treatment should be reported as an adverse event; including any pregnancies that occur in the partner of a study patient. Abortion,

`

whether accidental, therapeutic, or spontaneous, should be reported as an SAE. Congenital anomaly or birth defects, as defined by the 'serious' criterion above (see definitions Section 8.1) should be reported as an SAE

All pregnancies will be monitored for the full duration; all perinatal and neonatal outcomes should be reported. Infants should be followed for a minimum of 8 weeks.

8.3 Reporting Periods and Follow-Up for Adverse Events and Serious Adverse Events

Report all AEs and SAEs from the time of informed consent through the EOT visit or 30 days after the last study treatment, whichever is later. All SAEs that occur after the 30-day safety reporting period and are considered pertinent in the opinion of the Investigator must also be reported to the sponsor.

SAEs will be followed until significant changes return to baseline, the event stabilizes or is no longer considered clinically significant by the Investigator, or the patient dies or withdraws consent. All non-serious AEs will be followed through the EOT visit or 30 days after the last study treatment, whichever is later. Adverse events of peripheral neuropathy will be followed until they return to baseline or Grade 1, the patient dies or withdraws consent, or the study is closed. Certain other adverse events of interest may be followed until resolution, return to baseline, or study closure.

8.4 Immediate Reporting of Serious Adverse Events

All serious adverse events that occur during the study, regardless of the relationship to the study drug, must be reported by the Investigator to the following parties within 24 hours of being made aware of the SAE:

- The Sponsor-Investigator
- The Institutional Review Board (IRB) in accordance with institutional guidelines for reporting SAEs.
- Seattle Genetics Inc, the manufacturer of brentuximab vedotin, by faxing the SAE Report form and any copies of the relevant source documentation (e.g., hospital admission or discharge summary, laboratory or other test results, etc) that pertain to the event to: Seattle Genetics Drug Safety Department at 425-527-4308
- Cephalon

For initial SAE reports, available case details are to be recorded on an SAE form. At a minimum, the following should be included:

- Patient number
- Date of event onset
- Description of the event
- Study treatment, if known

`

The Institution shall notify the IRB and Cephalon within (1) business day, by facsimile, upon learning of the occurrence during the study of:

- All SAE/AE as described in Section 8.1.2 regardless of causality
- Any exposure of a pregnant study patient to the study drug within thirty (30) days of exposure
- Any female partner of a male study participant becoming pregnant within thirty (30) days of exposure
- Any medical event which may reasonably be believed to impair the integrity, validity, or ongoing viability of the study

Relevant follow-up information is to be submitted to the Sponsor-Investigator to Seattle Genetics (at the fax number above) and to Cephalon using the SAE transmittal form provided by Cephalon as soon as it becomes available.

In the event that the IRB requests additional safety information from Sponsor-Investigator, Sponsor-Investigator shall notify Cephalon of such request within one (1) business day.

8.5 IND Safety Reports

During the course of the study, the Sponsor may determine that certain safety reports are required to comply with regulations. The Sponsor is responsible for submission of such reports to their IND. Seattle Genetics is responsible for meeting their reporting requirements. The Investigator may receive a letter called an "IND Safety Report" from this study and/or other Seattle Genetics' sponsored studies (cross reports). These reports must be submitted to the IRB/EC as required.

9.0 DATA QUALITY CONTROL AND DATA ASSURANCE

9.1 Monitoring

The Institutional Review Board (IRB) at the Columbia University Medical Center will monitor this study.

9.1.2 RESPONSIBILITY FOR DATA SUBMISSION

The Columbia University Medical Center will act as the coordinating center. The study coordinator at CUMC is responsible for compiling data for all participants at CUMC. Study coordinators at the other two clinical sites are responsible for compiling the data for all participants at their sites, and providing this data to the CUMC study coordinator, and to the Principle Investigator for review.

9.2 Data Safety Monitoring Board

CUMC will act as the coordinating center. The HICCC NCI-approved Data Safety Monitoring Committee

`

(DSMC) will oversee the conduct of this trial. This protocol will adhere to the policies of the HICCC Cancer Center Data and Safety Monitoring Plan, version 2 guidelines in accordance with NCI regulations. The committee is led by Dr. Gregory Mears and consists of HICCC members. The DSMC meets monthly to review adverse event reporting and the timeliness of adverse event reporting. The PI will submit data and safety monitoring reports to the DSMC. Data from all 3 sites will be compiled into 1 report and all safety data will be reviewed here at CUMC.

Detailed guidelines regarding the structure, function and decision-making mechanisms for the Data Safety Monitoring Board are provided in the DSMB charter.

To ensure accurate, complete, and reliable data the Sponsor/Investigator will do the following:

- Provide instructional materials to the study sites as appropriate
- Instruct the investigators and study personnel on the protocol, the completion of CRFs, and study procedures
- Make periodic visits to the study site(s)
- Be available for consultation and stay in contact with the study site personnel by email, mail, telephone, or fax
- Monitor the patient data recorded in the CRF against source documents at the study site
- Review and evaluate the CRF data and use standard computer edits to detect errors in data collection
- The study may be audited by the Investigator/Sponsor, Seattle Genetics, or their representatives and/or regulatory agencies at any time. If contacted by a regulatory agency for an audit, the Investigator/Sponsor's study manager will be notified immediately. Contact information for the Investigator/Sponsor's study manager is included in the investigator file.

10.0 DATA ANALYSIS METHODS

10.1 Study Design

In phase 1, dose escalation will proceed according to a standard 3 + 3 dose escalation design. Three patients will be assigned to the starting dose level 1. If no DLT is observed, the trial proceeds to the next dose level and another cohort of 3 patients is enrolled. If 1 patient in a cohort experiences a DLT, then the cohort is expanded to 6 patients. If 2 of 3 or more patients in a cohort experience a DLT then the cohort will be designated the maximum administered dose (MAD) and the dose level below will be expanded. If no more than 2 of 6 patients experience a DLT, this dose level will be designated the MTD. If none of the additional patients experience a DLT (1 out of 6) then dose escalation continues. The MTD is defined as the highest dose level at which <33% of the dose cohort (0 of 3 or 1 of 6) experience a DLT in the first cycle of therapy. Up to a maximum of 10 patients will be added at the MTD to more fully characterize the safety of the drug combination. As long as <33% of patients experience a DLT, this dose cohort will remain the MTD.

For Phase 2, a two-stage Simon Minimax design, with a first stage

`

that includes 18 patients and a total of 37 patients in the two stages will test the hypothesis that the ORR is less than or equal to 0.50 versus the alternative that the ORR is greater than or equal to 0.75. If the combination is actually not effective, there is a 0.05 probability of incorrectly concluding that the combination is effective (Type I error rate). If the combination is actually effective, there is over 90% chance of correctly concluding that it is effective (power). With 18 patients studied in the first stage, the trial will be terminated if there are 9 or fewer responses. If the trial proceeds to the second stage, a total of 37 patients will be studied. If there are 23 or fewer responses the combination will be rejected for further study (calculations from PASS 2008, NCSS, J Hintze, Kaysville, UT). Disease and patient characteristics at baseline will be summarized using descriptive statistics. For qualitative variables, frequency distributions and proportions will be estimated; for quantitative variables, summary statistics (mean, median, standard deviations, range) and graphical displays (boxplots) will be used.

10.1.1 Sample Size

If all planned cohorts are treated, and the final dose level (dose level 5) is declared the MTD this study will treat 22 patients at phase 1. If all planned cohorts are expanded at phase 1 and the final cohort reached this study will treat up to 12 additional patients at phase 1. If MTD is reached earlier, fewer than 22 patients will be treated at phase 1. The phase 2 component consists of a maximum of 37 patients. This study is estimated to treat 59 patients, and at maximum will treat 71 patients.

10.2 General Statistical Considerations

Disease and patient characteristics at baseline will be summarized using descriptive statistics. For qualitative variables, frequency distributions and proportions will be provided; for quantitative variables, summary statistics (eg, mean, median, quartiles, standard deviations, etc) and graphical displays (eg, boxplots).

In phase 2 ORR will be estimated upon completion of the study along with exact 95% confidence intervals. Disease response will be assessed by CT or PET/CT according to the Revised Response Criteria for Malignant Lymphoma (33) after cycle 3 and after cycle 6. Patients who have stable disease or better after cycle 3 will continue through cycle 6. Response will be scored for best response.

Levels of peripheral cytokines and chemokines will be compared between overall responders and non-responders using the two-sample T-test or Wilcoxon rank sum test depending on the distribution of the data. Multivariable logistic regression models with response as the outcome will also be fit to the data to estimate adjusted odds ratios and identify the combination of independent variables most predictive of response. Variables which are associated with response in bivariate analyses as well those deemed to be important a priori based on clinical factors will be considered for inclusion in the model. An internal validation of the final predictive model will be performed using a 5-fold cross-validation procedure in which the model will be re-fit at each step of the validation using the training data (80% of the data), and evaluated on the corresponding test

`

set (remaining 20% of the data). Results of the validation procedure will summarized as the sensitivity, specificity, and accuracy of the model averaged over all 5 tests. We expect a minimum sample size of 40 subjects from the Phase 1/2 studies of Aim 1 to be available for the biomarker studies of Aim 2 and a response rate of 75%, yielding at least 30 responders and 10 non-responders. Based on the data from Gaiolla et al. and Weirauch et al., we estimated standard deviations for IL-6, IL-10, and TARC on the log scale of 0.83, 0.38, and 1.2 pg/ml, respectively, assuming the log-transformed values are normally distributed. Under these assumptions, the study will have 80% power to detect a 2.5 fold difference in IL-6, a 1.5 fold difference in IL-10 levels, and a 3.7 fold difference in TARC between responders and non-responders. These differences are similar in magnitude to the 2.2 fold and 1.8 fold differences in IL-6 and IL-10, respectively, which Gaiolla et al. observed between treatment failures and non-failures in HL patients and the 3.1 fold difference Weirauch et al. observed in TARC levels at baseline between HL patients with a continuous complete response after therapy versus progressive disease.

10.3 Patient Characteristics

Disease and patient characteristics at baseline will be summarized using descriptive statistics

10.4 Analysis Populations

All patients receiving at least one dose of study drug will be evaluable for toxicity. All patients must complete at least one cycle of therapy to be evaluable for efficacy.

10.5 Handling of Missing Data

Analyses will be based on observed data without imputation. Patients with missing values of a variable other than the time-to-event endpoints PFS and OS will be excluded from the analysis of that endpoint. Censoring rules will be applied to estimation of the distribution of the time-to-event endpoint.

10.6 Safety Analysis

10.6.1 Extent of Exposure

The dose (mg/kg) of study drugs administered, the total number of doses of study drugs, and the duration of treatment (number of study cycles) will be summarized with descriptive statistics. The number and percentage of patients whose dose is modified at any time will be summarized by each type of modification by cycle and overall. The proportion of patients completing each cycle of treatment will be summarized.

10.6.2 Dose Limiting Toxicity

Dose Limiting Toxicities will be evaluated during Cycle 1. For description of DLTs see section 5.7. Toxicities will be described by intensity at each dose level.

10.6.3 Adverse Events

Adverse events will be classified by system organ class and the preferred term using the Medical Dictionary for Regulatory Activities (MeDRA) and graded using NCI CTCAE Version 4.

`

The patient incidence of AEs will be summarized by treatment group, system organ class, preferred term, severity, and relationship to study drug. The relationship to study drug will be classified as “related” or “unrelated”. All AEs will be listed with the pertinent patient information.

Adverse events leading to dose modification or patient withdrawal will be summarized and listed in the same manner.

10.6.4 Deaths and Serious Adverse Events

Serious Adverse Events will be listed and summarized in a similar manner to AEs. Events with a fatal outcome will be listed.

10.7 Clinical Laboratory Results

Summary statistics for actual values and for changes from baseline will be tabulated for laboratory results by scheduled visit. Patients with laboratory values outside of the normal reference range at any post-baseline assessment will be summarized, and graded per NCI CTCAE Version 4 when applicable. Patient incidence of laboratory toxicity will be summarized by treatment group and maximum grade for each laboratory test.

10.8 Other Safety Analysis

Patients ECOG score will be recorded with each study visit, and tracked longitudinally until the EOT visit. Shifts from baseline to worst post-baseline score will be tabulated by treatment group. Patients who have ECOG scores that worsen post-baseline compared to baseline will be listed with other pertinent patient information.

10.9 Efficacy Analysis

The primary objective in phase 2 is to determine the ORR for the combination of brentuximab vedotin and bendamustine. Overall response rate (ORR) (PR+CR) is defined by the guidelines of the International Harmonization Project Group 2007 Revised Response Criteria, and will be tabulated by dose level. Response rates will be based on all patients who receive at least one cycle of therapy and complete a follow-up evaluation including PET/CT or CT scan.

In phase 1 ORR will be summarized descriptively. In phase 2 ORR will be estimated upon completion of the study along with exact 95% confidence intervals. With 37 patients in phase 2, the width of the exact 95% interval will range from 0.29 if the sample ORR is 0.75 to 0.32 if the sample ORR is 0.50.

10.10 Analysis of Secondary Endpoints

The number of dose delays >7 days will be described at the MTD.

`

Progression free survival is defined as the time from study entry to the first documentation of tumor progression or death due to any cause whichever comes first. Kaplan-Meir methodology will be used to summarize estimate median PFS for patients in all treatment groups.

Duration of response is defined as the time from documentation of a response to treatment to the first documentation of documentation of tumor progression or death due to any cause whichever comes first. Kaplan-Meir methodology will be used to summarize estimate median DOR for patients in all treatment groups.

Patients who undergo autologous or allogeneic BMT after the completion of study will be censored for PFS and DOR follow-up at the time of transplantation.

Overall survival will be described as the time from randomization to the date of death due to any cause. In the absence of confirmation of death, survival time will be censored from the last date the patient is known to be alive.

10.11 Analysis of Correlative Studies

For the 3 correlative endpoints, changes in the serum levels of Tarc, the peripheral blood lymphocyte expression of PD-1, and changes in the serum levels of IL-6 and IL-10 will be described and correlated with response to therapy.

For each of the correlative endpoints under study, measurements will be obtained pretreatment, during treatment and after treatment. Mixed effects logistic regression models that include time as a fixed effect and patient as a random effect will be used to examine the relationship of response to the changes over time in these parameters. These models take into account the repeated observations within a patient and can incorporate covariates as well as missing observations (Fitzmaurice, G., Laird, N. and Ware, J. Applied Longitudinal Analysis. John Wiley and Sons, 2004). The joint effects of TARC, PD-1, IL-6 and IL-10 changes over time as a predictor of response outcome will also be examined.

11.0 Informed Consent

11.1 Study Informed Consent

Study personnel must obtain documented consent from each potential patient prior to entering them in the clinical study. Consent must be documented on the IRB approved consent form by obtaining the dated signature both of the patient and of the investigator conducting the consent discussion. If the patient is unable sign the consent form, then oral consent, attested to by the dated signature of an impartial witness (someone not involved with the conduct of the study), is the required alternative.

If the patient is illiterate, an impartial witness should be present during the entire informed consent

`

reading and discussion. Afterward, the patient should sign and date the informed consent, if capable. The impartial witness should also sign and date the informed consent along with the individual who read and discussed the informed consent (i.e., study staff personnel).

If the patient is legally incompetent (i.e., a minor or mentally incapacitated), the written consent of a parent, legal guardian or legal representative must be obtained. An impartial witness will also sign such consent.

The information from the consent form should be translated and communicated to the subject in language understandable to the subject. Consent forms will be available in English and Spanish. When the study participant is non-English and non-Spanish speaking, the consent form must be read accurately in its entirety by a qualified professional translator. The translator will provide a written statement indicating that the consent form has been accurately translated from the accompanying English version, and that the study participant a consents to participation. The professional translator will sign the consent form as an impartial witness.

A copy of the signed and dated consent form should be given to the patient before participation in the study.

Patients may undergo study screening tests prior to giving written informed consent provided that these tests are considered part of standard care.

The initial informed consent form and any subsequent revised written informed consent form, and written information will receive the IRB approval. The patient or his/her legally acceptable representative will be informed in a timely manner if new information becomes available that may be relevant to the patient’s willingness to continue participation in the trial. The communication of this information will be documented.

12.0 References

1. Ries, LA, Kosary, CL, Hankey, BF, et al. (Eds). SEER cancer statistics review: 1973-1994, NIH publ no. 97-2789, National Cancer Institute, Bethesda 1997.
2. American Cancer Society (2009a). Cancer facts and Figures 2009. Available at: http://www.cancer.org
3. Canadian Cancer Society/National Cancer Institute of Canada (2009). Canadian Cancer Statistics 2009. Available at: http://www.cancer.ca
4. Longo DL, Duffey PL, Young RC, Hubbard SM, Ihde DC, Glatstein E, et al. Conventional-dose salvage combination chemotherapy in patients relapsing with Hodgkin's disease after combination chemotherapy: the low probability for cure. J Clin Oncol. 1992;10(2):210-8.
5. Urba WJ, Longo DL. Hodgkin's disease. N Engl J Med. 1992;326(10):678-87.
6. Bonfante V, Santoro A, Viviani S, Devizzi L, Balzarotti M, Soncini F, et al. Outcome of patients with Hodgkin's disease failing after primary MOPP-ABVD. J Clin Oncol. 1997;15(2):528-34.
7. Moskowitz CH, Kewalramani T, Nimer SD, Gonzalez M, Zelenetz AD, Yahalom J. Effectiveness of high dose chemoradiotherapy and autologous stem cell transplantation for patients with biopsy-proven primary refractory Hodgkin's disease. Br J Haematol. 2004;124(5):645-52.
8. Stein H, Foss HD, Durkop H, Marafioti T, Delsol G, Pulford K, et al. CD30(+) anaplastic large cell lymphoma: a review of its histopathologic, genetic, and clinical features. Blood. 2000;96(12):3681-95.
9. Jacobsen E. Anaplastic large-cell lymphoma, T-/null-cell type. Oncologist. 2006;11(7):831-40.
10. Tilly H, Gaulard P, Lepage E, Dumontet C, Diebold J, Plantier I, et al. Primary anaplastic large-cell lymphoma in adults: clinical presentation, immunophenotype, and outcome. Blood. 1997;90(9):3727-34.
11. American Cancer Society (2008). Facts and figures. Available at: http:www.cancer.org
12. Wilson WH, Bryant G, Bates S, Fojo A, Wittes RE, Steinberg SM, et al. EPOCH chemotherapy: toxicity and efficacy in relapsed and refractory non-Hodgkin's lymphoma. J Clin Oncol. 1993;11(8):1573-82.
13. Velasquez WS, McLaughlin P, Tucker S, Hagemeister FB, Swan F, Rodriguez MA, et al. ESHAP--an effective chemotherapy regimen in refractory and relapsing lymphoma: a 4-year follow-up study. J Clin Oncol. 1994;12(6):1169-76.
14. Gardner LJ, Polski JM, Evans HL, Perkins SL, Dunphy CH. CD30 expression in follicular lymphoma. Arch Pathol Lab Med. 2001;125(8):1036-41.
15. Horie R, Watanabe T. CD30: expression and function in health and disease. Semin Immunol. 1998;10(6):457-70.
16. Pizzolo G, Romagnani S. CD30 molecule (Ki-1 Ag): more than just a marker of CD30+ lymphoma. Haematologica. 1995;80(4):357-66.
17. Younes A, Carbone A. CD30/CD30 ligand and CD40/CD40 ligand in malignant lymphoid disorders. Int J Biol Markers. 1999;14(3):135-43.
18. Sutherland MS, Sanderson RJ, Gordon KA, Andreyka J, Cerveny CG, Yu C, et al. Lysosomal trafficking and cysteine protease metabolism confer target-specific cytotoxicity by peptide-linked anti-CD30-auristatin conjugates. J Biol Chem. 2006;281(15):10540-7.
19. Francisco JA, Cerveny CG, Meyer DL, Mixan BJ, Klussman K, Chace DF, et al. cAC10-vcMMAE, an anti-CD30-monomethyl auristatin E conjugate with potent and selective antitumor activity. Blood. 2003;102(4):1458-65.
20. Younes A, Bartlett NL, Leonard JP, Kennedy DA, Lynch CM, Sievers EL, et al. Brentuximab vedotin (SGN-35) for relapsed CD30-positive lymphomas. N Engl J Med. 2010;363(19):1812-21.

`

21. Knauf WU, Lissichkov T, Aldaoud A, Liberati A, Loscertales J, Herbrecht R, et al. Phase III randomized study of bendamustine compared with chlorambucil in previously untreated patients with chronic lymphocytic leukemia. J Clin Oncol. 2009;27(26):4378-84.
22. Robinson KS, Williams ME, van der Jagt RH, Cohen P, Herst JA, Tulpule A, et al. Phase II multicenter study of bendamustine plus rituximab in patients with relapsed indolent B-cell and mantle cell non-Hodgkin's lymphoma. J Clin Oncol. 2008;26(27):4473-9.
23. Rummel MJ, Al-Batran SE, Kim SZ, Welslau M, Hecker R, Kofahl-Krause D, et al. Bendamustine plus rituximab is effective and has a favorable toxicity profile in the treatment of mantle cell and low-grade non-Hodgkin's lymphoma. J Clin Oncol. 2005;23(15):3383-9.
24. Rummel, M. American Society of Hematology. 2009. Abstract 720.
25. Borchmann, P, Schnell, R. Diehl V. Engert, A. New Drugs in the Treatment of Hodgkin's Disease. Ann Oncol 1998;9 Suppl 5:S103-8.
26. Moskowitz A, ASH Abstract 2009
27. Moskowitz C. Risk-adapted therapy for relapsed and refractory lymphoma using ICE chemotherapy. Cancer Chemother Pharmacol. 2002;49 Suppl 1:S9-12.
28. Aldinucci D, Gloghini A, Pinto A, De Filippi R, Carbone A. The classical Hodgkin's lymphoma microenvironment and its role in promoting tumour growth and immune escape. J Pathol. 2010;221(3):248-63.
29. Buglio D, Georgakis GV, Hanabuchi S, Arima K, Khaskhely NM, Liu YJ, et al. Vorinostat inhibits STAT6-mediated TH2 cytokine and TARC production and induces cell death in Hodgkin lymphoma cell lines. Blood. 2008;112(4):1424-33. PMCID: 2515130.
30. Yamamoto R, Nishikori M, Kitawaki T, Sakai T, Hishizawa M, Tashima M, et al. PD-1-PD-1 ligand interaction contributes to immunosuppressive microenvironment of Hodgkin lymphoma. Blood. 2008;111(6):3220-4.
31. Green MR, Monti S, Rodig SJ, Juszczynski P, Currie T, O'Donnell E, et al. Integrative analysis reveals selective 9p24.1 amplification, increased PD-1 ligand expression, and further induction via JAK2 in nodular sclerosing Hodgkin lymphoma and primary mediastinal large B-cell lymphoma. Blood. 2010;116(17):3268-77. PMCID: 2995356.
32. Gaiolla RD, Domingues MA, Niero-Melo L, de Oliveira DE. Serum levels of interleukins 6, 10, and 13 before and after treatment of classic hodgkin lymphoma. Arch Pathol Lab Med. 2011;135(4):483-9.
33. Cheson BD, Pfistner B, Juweid ME, Gascoyne RD, Specht L, Horning SJ, et al. Revised response criteria for malignant lymphoma. J Clin Oncol. 2007;25(5):579-86.
34. Herold M, Schulze A, Niederwieser D, Franke A, Fricke HJ, Richter P, et al. Bendamustine, vincristine and prednisone (BOP) versus cyclophosphamide, vincristine and prednisone (COP) in advanced indolent non-Hodgkin's lymphoma and mantle cell lymphoma: results of a randomised phase III trial (OSHO# 19). J Cancer Res Clin Oncol. 2006;132(2):105-12.

`

13.0 Appendices

Appendix 1: Dosing of TREANDA for Injection in Obese Patients

Dosing of TREANDA® (bendamustine HCl) for Injection in Obese Patients

No formal pharmacokinetic or pharmacodynamic studies have been conducted to determine whether dose adjustment is necessary for dosing of TREANDA in obese patients (eg, when calculating the body surface area [BSA] required). Summarized below for your review is information from the prescribing information on the recommended dosing of TREAND in patients with chronic lymphocytic leukemia (CLL) and indolent B-cell non-Hodgkin's lymphoma (NHL), and available data from the clinical program for TREANDA on dosing in obese patients.

Data from the Prescribing Information[1]

Recommended Dosage

Dosing Instructions for CLL

The recommended dose is 100 mg/m^2 administered intravenously over 30 minutes on Days 1 and 2 of a 28-day cycle, up to 6 cycles.

Dosing Instructions for NHL

The recommended dose is 120 mg/m^2 administered intravenously over 60 minutes on Days 1 and 2 of a 21-day cycle, up to 8 cycles.

Additional Information

No formal pharmacokinetic or pharmacodynamic studies have been conducted to the necessity of adjusting the dosing weight (actual vs ideal) of obese patients when calculating the body surface area (BSA) required for TREANDA dosing. Cephalon has no specific recommendations regarding the adjustment of dosing for obese patients nor does Cephalon have any recommendations regarding a suggested maximum BSA when dosing TREANDA.

In the pivotal, bendamustine CLL trial, the mean weight for patients in the bendamustine group was 78.2 kg (range, 50-133 kg), and the mean BSA was 1.9 m^2 (range 1.5-2.4 m^2). In the pivotal NHL trial, the mean weight for those patients administered bendamustine was 86.7 kg (range, 44-151 kg), and the mean BSA was 2.0 m^2 (range, 1.3-2.7 m^2). The protocols for these studies did not specify dosing weight adjustments or a maximum BSA when dosing bendamustine in obese patients.[2,3,4,5,6]

In an internal retrospective review of the bendamustine CLL and NHL study data, the actual administered dose in both obese and non-obese groups was generally close to the calculated dose based on actual body weight.[5,6] There did not appear to be a signal of increased adverse events in the obese patients treated with TREANDA whose dose was based on actual body weight.

`

Summary

No formal pharmacokinetic or pharmacodynamic studies have been conducted to determine whether dose adjustment is necessary for dosing of TREANDA in obese patients (eg, when calculating the body surface area [BSA] required). In clinical studies the actual administered dose in both obese and non-obese groups was generally close to the calculated dose based on actual body weight, and there did not appear to be an increase in the occurrence of adverse events observed in obese patients compared to non-obese patients. Cephalon has no specific recommendations regarding dose adjustment of TREANDA for obese patients.

For prescribing information, please see the enclosed package insert for TREANDA.

---

[1] TREANDA (bendamustine HCl) for Injection [current approved prescribing information]. Frazer, PA: Cephalon, Inc.

[2] Knauf WU, Lissichkov T, Aldaoud A, et al. Phase III randomized study of bendamustine compared with chlorambucil in previously untreated patients with chronic lymphocytic leukemia. *J Clin Oncol*. 2009; 27:4378-4384.

[3]Knauf WU, Lissichkov T, Aldaoud A et al. Bendamustine versus chlorambucil in treatment-naive patients with B-cell chronic lymphocytic leukemia (B-CLL): results of an international phase III study [abstract 2043] [Poster Presentation 233-II]. Presented at the American Society of Hematology 49th Annual Meeting, December 8-11, 2007, Atlanta, Georgia.

[4]Kahl B, Bartlett NL, Leonard JP et al. Bendamustine is effective therapy in patients with rituximab-refractory, indolent b-cell non-hodgkin lymphoma. *Cancer*. 2010;116:106-114.

[5] Data on file (Bendamustine Study 02CLLIII). Frazer, PA: Cephalon, Inc.

[6] Data on file (Bendamustine Study SDX 105-03). Frazer, PA: Cephalon, Inc
